# Intervention Use and Action Planning in a Web-Based Computer-Tailored Weight Management Program for Overweight Adults: Randomized Controlled Trial

**DOI:** 10.2196/resprot.2599

**Published:** 2014-07-23

**Authors:** Lenneke van Genugten, Pepijn van Empelen, Anke Oenema

**Affiliations:** ^1^Erasmus University Medical CenterDepartment of Public HealthRotterdamNetherlands; ^2^Research Group Life StyleTNOLeidenNetherlands; ^3^Department of Health PromotionCAPHRI School for Public Health and Primary CareMaastricht UniversityMaastrichtNetherlands

**Keywords:** behavior change, obesity, health promotion, intervention research

## Abstract

**Background:**

There are many online interventions aiming for health behavior change but it is unclear how such interventions and specific planning tools are being used.

**Objective:**

The aim of this study is to identify which user characteristics were associated with use of an online, computer-tailored self-regulation intervention aimed at prevention of weight gain; and to examine the quality of the goals and action plans that were generated using the online planning tools.

**Methods:**

Data were obtained with a randomized controlled effect evaluation trial in which the online computer-tailored intervention was compared to a website containing generic information about prevention of weight gain. The tailored intervention included self-regulation techniques such as personalized feedback, goal setting, action planning, monitoring, and other techniques aimed at weight management. Participants included 539 overweight adults (mean age 46.9 years, mean body mass index [BMI] 28.03 kg/m^2^, 31.2% male, 11% low education level) recruited from the general population. Use of the intervention and its planning tools were derived from server registration data. Physical activity, fat intake, motivational factors, and self-regulation skills were self-reported at baseline. Descriptive analyses and logistic regression analyses were used to analyze the results.

**Results:**

Use of the tailored intervention decreased sharply after the first modules. Visiting the first tailored intervention module was more likely among participants with low levels of fat intake (OR 0.77, 95% CI 0.62-0.95) or planning for change in PA (OR 0.23, 95% CI 0.05-0.97). Revisiting the intervention was more likely among participants high in restrained eating (OR 2.45, 95% CI 1.12-5.43) or low in proactive coping skills for weight control (OR 0.28, 95% CI 0.10-0.76). The planning tools were used by 5%-55% of the participants, but only 20%-75% of the plans were of good quality.

**Conclusions:**

This study showed that psychological factors such as self-regulation skills and action planning were associated with repeated use of an online, computer-tailored self-regulation intervention aimed at prevention of weight gain among adults being overweight. Use of the intervention was not optimal, with a limited number of participants who visited all the intervention modules. The use of the action and coping planning components of the intervention was mediocre and the quality of the generated plans was low, especially for the coping plans. It is important to identify how the use of action planning and coping planning components in online interventions can be promoted and how the quality of plans generated through these tools can be improved.

**Trial Registration:**

Netherlands Trial Register: NTR1862; http://www.trialregister.nl/trialreg/admin/rctview.asp?TC=1862 (Archived by WebCite at http://www.webcitation.org/6QG1ZPIzZ).

## Introduction

### Overview

Considering the lack of effective long-term treatments for obesity, prevention of obesity is very important [1,2]. This can be achieved by prevention of weight gain. This is particularly important among people who are overweight (body mass index [BMI] 25-30 kg/m^2^) because they are most at risk of becoming obese. The Internet may be a relevant medium to reach the large group of overweight people.

The Internet is increasingly being used as a channel for the delivery of interactive and individualized interventions to promote healthy lifestyles among various populations [3-6]. Such interventions can be effective at improving a variety of behaviors and outcomes [7-10], especially when a planning tool is included [11]. However, a large body of evidence suggests that the use of online interventions is often low [12-14]. In this paper, we focused on the use of GRIPP, which is an online computer-tailored self-regulation program aimed to prevent weight gain among overweight (BMI 25-30 kg/m^2^) adults [15]. The computer-tailored intervention consisted of 4 modules that people could visit in a 4- to 8-week period. Although this intervention did not show an additional effect over generic information as far as improving BMI, waist circumference, skinfold thickness, physical activity, and dietary intake, this result may in part be due to implementation failures. Various authors have suggested that process evaluations aimed at studying the efficacy of the implementation process are vital to optimize interventions and to ensure an actual effect (eg, [16]).

Similar to the GRIPP study [17], a steep decline in numbers of visitors to follow-up sessions is often observed, and nonoptimal use or exposure to the intervention content may result in an underestimation of the effects that can be achieved with an online intervention [18]. More evidence is needed with regard to implementation factors that may be associated with intervention use [16,19], such as dose and fidelity [12,18,20]. Using the GRIPP study, we systematically examined two implementation aspects. The first aim was to identify factors that are associated with first and repeated use of an online weight gain prevention program for overweight adults (ie, dose delivered). The second aim was to increase insight into the amount and quality of use of the planning tools in the online interventions (ie, fidelity).

### Determinants of Intervention Use

To understand the potential impact that an intervention may have, it is important to understand who is reached by the intervention, when people are likely to engage in intervention activities and continue engaging in these activities and the extent to which the intervention is used as planned. Such factors may help to understand program implementation (failure) as well as ways to improve the quality of implementation [16,19].

The existing literature suggests that intervention use may be related to individual (ie, age, sex, education, BMI), motivational, and behavioral factors. Older adults were more likely to use online interventions [12,14], and women [12-14] have been found to be more likely to use online interventions, but the evidence is inconclusive with respect to the level of education. Visiting and revisiting an online intervention may be related to risk factors such as higher-than-recommended intake of saturated fat [12], elevated cholesterol level [13], and higher [14] or lower body weight [12]. Thus, several studies examined the use of online interventions, but the results were inconclusive. Furthermore, little is known about the influence of psychological traits. Therefore, the possible influence of psychological traits, such as weight locus of control, restrained eating, and self-regulation skills, in addition to more traditional predictors on online intervention use were studied.

The present intervention was developed based on the principles of self-regulation theory [21-23]. Key processes in self-regulation are goal selection, action (planning), and evaluation. Such an intervention may have more appeal to people who already embrace the concept of self-regulation because it fits them better. Furthermore, self-regulation skills, including planning and coping, may decrease the intention-behavior gap and increase the likelihood of actual performance of a desired behavior (eg, [24,25]). However, self-regulation skills are likely to be a generalized concept, indicating that people with more self-regulation skills for health-related behaviors may also be better at planning to visit or revisit an intervention, because this is also an example of behavior regulation. Therefore, we hypothesize that those high in baseline self-regulation skills are more likely to visit and revisit the intervention.

There are two other important factors that influence weight-related behavior and may also influence intervention use: weight locus of control and restrained eating. Weight locus of control refers to perceived control of one’s body weight. People who lack a feeling of control have been found to have less confidence in weight loss behaviors and a lower behavioral intention. Moreover, higher control is positively related to picking up weight loss ideas from an earlier intervention [26]. Therefore, we hypothesize that participants with a high locus of control are more likely to visit and revisit the intervention. For those with a more external locus of control, the intervention is probably less interesting, as they may not believe that it is possible to regulate their own behavior.

Previous studies have shown that restrained eating can be related to weight-related outcomes and participation among obese participants [27]. We hypothesize that restrained eaters are more likely to visit and revisit the intervention because they will use the opportunity to improve their control over their (eating) behavior and weight. A self-regulation intervention may thus be extra attractive to them.

### Quality of Use: Action and Coping Planning

Self-regulation often starts with goal selection—determining what one wants to achieve. This goal is the reference point for all other related activities, such as monitoring progress of behavior change toward the goal [22]. However, to serve as a useful reference point, the goal must be very specific (eg, indicating what will be done at what time). Action planning specifies where, when, and how to act [28]. Coping planning (ie, linking anticipated risk situations with a suitable coping response) is a recurring event in self-regulation, because it allows the person to adapt his or her behavior to change or unfavorable circumstances [23,25]. Therefore, goal selection, action planning, and coping planning were important intervention components [29-31]. Studying the use of these tools will tell us more about the fidelity of implementation of these tools [16].

Because action plans must be of good quality to be effective [32], we aimed to study the use and quality of the goals and plans made by the participants [11]. In this trial, a guided, open format was chosen. Insight into the quality of plans generated through this type of planning tool is highly relevant because it can help to improve online self-regulation interventions.

This trial aimed to answer two questions: (1) Which baseline demographic, psychological factors, behavioral factors, and self-regulation skills are associated with first time and repeated use of an online computer-tailored self-regulation intervention aimed at preventing weight gain among overweight adults? (2) Do participants use the guided, open format tools for action planning and coping planning, and if so, what is the quality of the generated plans?

## Methods

### Design, Participants, and Recruitment

The data for this study were generated in a randomized trial (NTR1862) to establish the effects of the intervention on anthropometric and behavioral outcomes. More information about this trial can be read in van Genugten [17]. In this trial, the tailored intervention website was compared to a generic information website. For this study, only data from the tailored intervention website was used. Anthropometrics and self-reported behavior were assessed at baseline and 6 months after the intervention.

Participants were recruited from the general population through advertisements placed in local newspapers and flyers that were distributed door-to-door, in the waiting rooms of general practitioners and among the employees of four large companies. Participants enrolled in the study by filling out an online submission form. Subsequently, criteria for inclusion (25-60 years of age, BMI 25-30 kg/m^2^, ability to read and write in Dutch, and easy access to the Internet) and exclusion criteria (pregnancy, following a diet prescribed by a dietician or physician, having a history of depression or eating disorder) were used. In total, 630 people completed the online registration, and 516 initially participated by completing the baseline questionnaire and/or coming in for anthropometric measurements (n=480). Two hundred sixty-nine participants were allocated to the tailored intervention group and were included in this trial.

### Procedures

After subscription, participants received a confirmation letter and information leaflet about the trial. They also received an email in which they were asked to fill out the online baseline questionnaire (motivational factors, dietary intake, physical activity, and self-regulation skills). Weight, height, waist circumference, and skinfold thickness were measured at the hospital site where they also filled out the informed consent form. Participants preferably completed both measurements (anthropometrics and questionnaire) but were randomized even if they had completed only one measurement.

All randomized participants received a login name and password by email to access their assigned intervention program. Participants were asked to (re)visit the website at least 3 or 4 times during a 2-month period. They received biweekly email reminders to (re)visit the intervention website. Six months after completion of the intervention period, participants were asked by email to fill out the online questionnaire again and their anthropometrics were assessed at the hospital site. Phone calls were made to participants who did not respond by email. Participants who filled out the questionnaire and had their anthropometrics measured at the 6-month follow-up received a gift voucher of €10 (US$13.65).

### The Intervention

#### Tailored Intervention

The intervention’s main objective was to prevent weight gain in overweight adults by inducing small changes (100 kcal/day) in energy balance-related behaviors. Examples of these changes include increasing the frequency and duration of physical activity and reducing the intake of calories from several categories, such as dairy, meat, cheese, sauce and gravy, snacks, and sweetened drinks [33]. The intervention goals, methods, and strategies were based on self-regulation theory [22], motivational theories [28,34,35], and goal-setting and action-planning theories [28,36].

The intervention consisted of 4 modules. To deliver the self-regulation strategies in a timely fashion, each module was to be visited one week after the previous one, guiding the participant through all steps of self-regulation (goal setting, active goal pursuit, and evaluation [22]). Completion of all modules would take about 90 minutes. The first module aimed at increasing participants’ commitment to prevent weight gain by first asking them to weigh the pros and cons of weight gain prevention, and to choose one behavior change and plan for that change. The second and third modules evaluated progress on behavior change by giving participants feedback on their performance during the previous week, based on self-reported behavior change. If necessary, the intervention supported adaptation of the action and coping plans (when the participants failed to achieve the behavioral goals). The 4th module instructed participants on how to maintain self-regulation of body weight without using the program and they were provided with a tool to monitor and evaluate changes in their body weight. Modules 1, 2, and 3 are each supposed to be used at least one week apart. As they use the modules, participants fill in their body weight every week. When using the 4th module, a graph is made, showing the weight development of the participants. Furthermore, written feedback is provided. Both the graph and feedback show the normal weight range of the participants (taking daily and weekly fluctuations into account), indicating when weight is actually gained or lost. To conclude the program, the participants sign a personalized contract, which includes the goals and plans they had written down in the intervention, as well as their weight status and information for weight regulation in the future.

**Figure 1:**
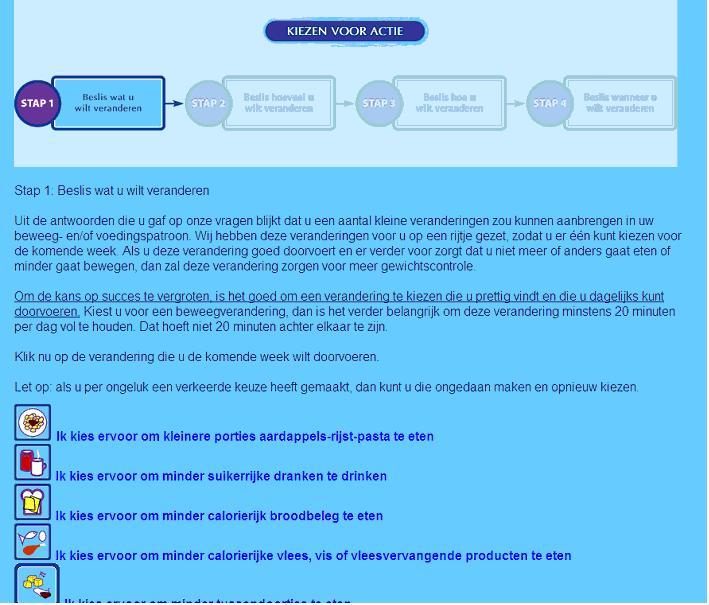
Goal setting and action planning in the GRIPP intervention.

**Figure 2:**
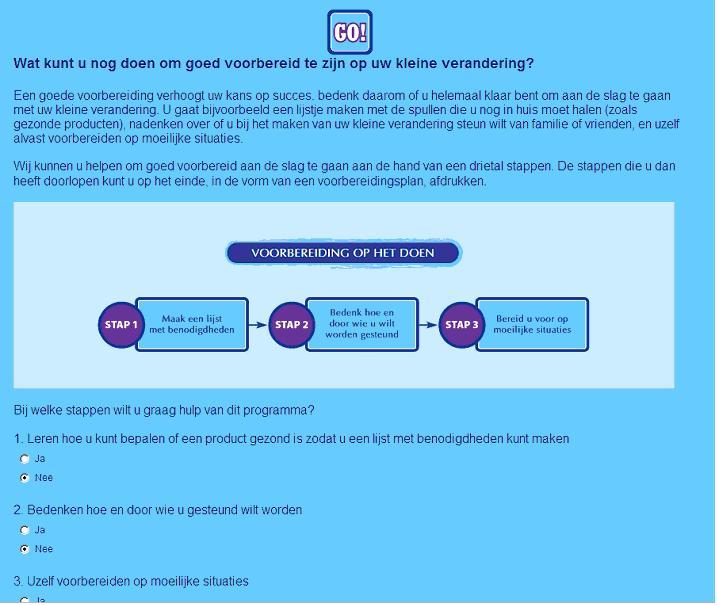
Coping planning in the GRIPP intervention.

The tailored modules were embedded in a website that also contained recipes, a peer-to-peer forum, and links to useful websites. Reminders to (re)visit the intervention were sent to the participants every two weeks. A more detailed description of the intervention’s contents can be found elsewhere [15]. Visuals of the intervention can be found in Figure 1, Figure 2, and Multimedia Appendix 1.

#### Action Planning

Based on the tailored feedback on dietary intake (DI) or physical activity (PA), people were guided in choosing what they wanted to change (goal setting) and where, when, and how to make the change (action planning) in an open format. The guided, open format was chosen to allow for personal preferences, which is important for motivation [37]. Moreover, this format was supposed to lead to very specific goals and plans. Such goals and plans usually have a positive influence on perceived behavioral control [38].

To establish a goal, people could first choose a category of change (eg, sweetened drinks or snacks) in which the feedback indicated that improvement was possible. Then, more specific feedback was provided on possible changes within the category. For example, first they could choose to decrease their snack intake. In the next step, they could choose what they want to eat (eg, fewer chocolate bars or salty snacks like peanuts). Participants were encouraged to choose a change that they would like to make and feel high self-efficacious about. Finally, participants had to fill out the content of the change, size of the change (eg, number of minutes of PA) and, if necessary, decide on an alternative (eg, eat an apple instead of a candy bar) to translate their goal into an action plan. For example, a correct action plan might be “If I have breakfast, I will eat 2 sandwiches (size) with nonfat cheese instead (alternative) of normal cheese (content).”

#### Coping Planning

To prevent relapse in the first week of change, people were asked whether they expected to encounter a risk situation (a situation in which they expected that making the change might be difficult; eg, at a party). If they did, they were asked to think about this situation and to describe their (coping planning) strategy to avoid or handle the situation. They could write down their strategy in text boxes [39] in a guided, open format. Together, the description of the situation and the strategy resulted in an implementation intention [38]. An example of a good coping plan would be “If my colleagues are eating pie and offer me a slice, I will say no and eat an apple.”

### Dependent Measures

#### Intervention Use

An objective measure of intervention use was obtained by retrieving the log-in data from the intervention server registrations, which registered how often each participant logged in to the program and which intervention modules they visited (0-3 for generic information, 0-4 for tailored intervention). First, a dichotomous “never-ever” score was created, with 0 indicating “never visited” and 1 indicating “visited at least once” (sum score >1). For those who visited at least 1 module (sum score >1), a dichotomous score was made for “revisiting” (visited first module only: 0, also visited later modules: 1). This categorization was based on the 3 steps of intervention use as defined by Brouwer et al [12,20]: landing at a website, visiting a website, and coming back for a second visit.

#### Use of Action-Planning and Coping-Planning Components and Quality of Goals and Plans

Information about the use of the action-planning and coping-planning components and the quality of the plans developed by the participants was also obtained from the intervention server registration, where the plans that had been written were stored. Two dichotomous variables were made, indicating whether people chose to make a change in dietary intake and/or physical activity. A dichotomous variable was created for use of the action-planning component (0: no plan, 1: a plan). Then, the quality of the goal was determined by scoring the text that was written in the text boxes in the program. For this text, 1 point was obtained if a challenging but realistic goal was stated (eg, increase walking by 30 minutes daily) and 1 point was obtained if the situation in which the change would be made was clearly and realistically stated (eg, when going to and returning from work). For PA, a third point could be obtained for filling out with whom one was planning to do the activity (eg, with my partner or alone). Therefore, 3 points could be obtained for a stated PA goal, and 2 points could be obtained for a DI goal.

A similar approach was used for use and quality of the coping plans, in particular how the participant planned to avoid or cope with a difficult situation in the first week of behavior change. A dichotomous variable was created based on the participant’s use of the coping-planning component (0: did not describe a coping plan, 1: described a coping plan). Next, the content of the coping plan was coded to assess its quality. A coping plan was coded as correct (scoring a 2) if a response was given that (1) would facilitate the desired behavior, and (2) was feasible in the risk situations that were defined [40]. If either or both of these criteria were not met, one point was given to indicate an incorrect plan.

All goals and coping plans were coded by 2 researchers (LVG and HVDP) separately, and then discussed until agreement was obtained.

### Independent Measures

#### Motivational Variables

Intention to prevent weight gain, perceived behavior control, weight locus of control, and restrained eating are potential determinants of intervention use and were assessed by online self-report at baseline. A description of the assessments of these factors is described in Multimedia Appendix 2.

Weight locus of control was assessed using a translation of the Weight Locus of Control scale [41], which has 4 statements (two externally and two internally oriented items). Factor analyses showed that only one factor could be identified. The scale reliability (Cronbach alpha) of the four items was 0.61, which is low, but comparable to the original scale [41]. Thus, a composite measure (mean value) was created.

Restrained eating was assessed with the restrained subscale of the Dutch Eating Behavior Questionnaire [42,43]. This questionnaire consists of 10 items about restrained eating. Cronbach alpha of all items was 0.87 and all items were combined to one mean value.

#### Self-Regulation Skills

Because monitoring weight, planning for PA, planning for DI, and proactive coping skills could be related to the participant’s use of the intervention, these can be considered intervention outcomes. These variables were assessed by self-report at baseline and at the 6-month follow-up. A description of the assessments of these factors is provided in Multimedia Appendix 2.

A dichotomous variable was made for monitoring of weight: weighing weekly (1) and not weighing weekly (eg, daily or never; 0).

Planning for PA was assessed with 4 items and planning for DI was assessed with 3 items. Cronbach alpha was 0.92 for planning for PA and 0.94 for planning for DI. Therefore, composite measures (mean scores) were calculated for PA and DI, respectively.

Proactive coping skills toward body weight were measured using the 21-item Proactive Competence Scale [44], which is based on the 5 phases of coping: (1) resource accumulation, (2) recognition of potential stressors, (3) initial appraisal, (4) preliminary coping efforts, and (5) elicitation and use of feedback concerning initial efforts [45]. All items were combined into one mean score, which had a Cronbach alpha of 0.92.

#### Fat Intake and Physical Activity

Fat intake and physical activity were assessed by self-report at baseline and 6 months after the intervention.

Fat intake was assessed using a food frequency questionnaire that assessed the frequency and quantity of a variety of high-energy food eaten in the past week. It was based on a Dutch validated questionnaire [46], and it enabled the researcher to calculate fat intake in fat points. The questionnaire consisted of 74 questions and was organized according to meal pattern. Participants recorded their frequency of consumption and portion size for a selection of food items eaten during meals or between meals. Higher scores indicate more frequent and/or larger amounts of fat intake. There were 23 products that fell into the following categories: dairy products (5), butter (1), gravy (1), sandwich fillings (3), meat and cheese for main dinner (2), and sweet, salty, hot and cold snacks (11 in total). In total, a maximum of 83 fat points could be obtained.

Physical activity was assessed using a questionnaire based on the Dutch validated Short QUestionnaire to ASsess Health-enhancing physical activity (SQUASH, developed to assess habitual physical activity) [47]. In this 16-item questionnaire, participants were asked to indicate how many days of the week they participated in specific activities and how much time they engaged in the activity per occasion. For active transport, respondents were asked how often they cycled and walked from home to work, and the duration. The same questions were asked about walking and cycling during leisure time. Furthermore, participants were asked how many different sports they did on a weekly basis (with a maximum of 4). For each different sport, they were asked to pick their sport activities (eg, swimming, running, soccer) from a list, indicating the weekly frequency and the average time they engaged in that activity per occasion. For each category, the mean number of minutes per day was calculated by multiplying the frequency with the duration and dividing this number by 7. Next, the total number of minutes engaged in physical activity per day was calculated as the sum of all activities (active transportation, leisure time activities, and sports).

#### Body Mass Index

The body measurements were performed by trained research assistants, following a measurement protocol. Participants’ height was measured twice at baseline using a Seca mobile height rod with an accuracy of 0.1 cm. The mean of both measures was used for height. A calibrated electronic digital floor scale (Seca 888 class III) was used to measure body weight, with an accuracy of 0.2 kg. The measures of height and weight were used to calculate BMI (weight [kg]/height [m]^2^). Body weight was measured at baseline and 6 months after the intervention period.

#### Sociodemographic Factors

Sex (male/female), date of birth, and educational level were assessed in the baseline questionnaire. To determine age, we asked participants their date of birth. Education was assessed by asking the participants to indicate what their highest completed level of education was (choosing 1 of 8 options). A 3-category variable was subsequently made, indicating a low (completed no education, primary school, secondary school, or lowest level of high school or lower vocational training), medium (completed intermediate or high level high school), or high (completed higher vocational training, college or university) level of education.

### Analyses

Descriptive statistics were used to describe the study population in terms of baseline demographic, behavioral, and psychological factors. Logistic regression analyses were applied to study participant predictors of first intervention visit and follow-up visits (dependent variables). To identify the best predictors of use, a backward elimination (likelihood ratio) procedure was used. Independent variables were age, education, sex, BMI, fat intake, physical activity, intention, and perceived behavioral control for weight gain prevention, weight locus of control, restrained eating, monitoring of weight, action planning for change in DI and PA, and proactive coping skills as assessed at baseline.

Descriptive statistics were used to describe the use of the self-regulation components and the quality of the participants’ plans.

## Results

### Study Population

The mean age of the participants was 47.7 years (SD 9.2), 31.3% (84/269) were male, 10.3% (24/232) had a low level of education, and 48.7% (113/232) had a medium level of education. The mean BMI was 28.1 kg/m^2^ (SD 2.02; Table 1).

**Table 1:** Baseline characteristics of the study participants.

Characteristics				Values
**Demographics** ^a^				
	Age (years), mean (SD)			47.7 (9.2)
	Male, n (%)			84/269 (31.2)
	**Education level, n (%)**			
		Low		24/232 (10.3)
		Medium		113/232 (48.7)
		High		95/232 (40.9)
**Outcome measures**				
	BMI, kg/m^2^, mean (SD)			28.17 (2.02)
	**BMI, n (%)**			
		Healthy weight		9/224 (4.0)
		Overweight		169/224 (75.4)
		Obese		46/224 (20.5)
	Fat intake, points, mean (SD)			17.02 (6.0)
	Physical activity, minutes, mean (SD)			63.1 (50.4)
**Motivational factors** ^b^				
	Intention for weight gain prevention, score (SD)			4.71 (0.6)
	Perceived behavioral control for weight gain prevention, score (SD)			4.3 (0.8)
**Self-regulation factors**				
	Weekly monitoring weight, n (%)^e^			112/230 (48.7)
	**Action planning,** ^c^ **mean (SD)**			
		DI		2.30 (1.0)
		PA		2.08 (1.0)
	Proactive coping skills,^c^mean (SD)			2.67 (0.5)
	Weight locus of control,^c^mean (SD)			3.76 (0.66)
	Restrained eating,^b^mean (SD)			3.11 (0.63)

^a^N values are based on number of respondents.

^b^Score range 1-5.

^c^Score range 1-4.

### Intervention Use

The first intervention module was visited by 93.3% (251/269) of the participants (Figure 1), the second by 74.1% (199/269), the third 26.7% (71/269), and the fourth and last module by 15.2% (40/269). The mean number of visits was 1.8 and the median was 1. Logistic regression analysis (Table 2) showed that those with a lower level of physical activity (odds ratio [OR] 0.98, 95% CI 0.96-0.999), lower action planning for PA (OR 0.23, 95% CI 0.06-0.9) and lower fat intake (OR 0.77, 95% CI 0.62-0.95) at baseline were more likely to visit the intervention once.

Those with low proactive coping skills (OR 0.28, 95% CI 0.10-0.76) and high levels of restrained eating were more likely to revisit the intervention (OR 2.45, 95% CI 1.11-5.43).

**Table 2:** Results of multivariable backward logistic regression analyses examining potential correlates of use and repeated use of the tailored intervention (N=269).

Predicting factors			Using the intervention at least once	Using the intervention at least twice
			OR (95% CI)^a^	OR (95% CI)
**Demographic factors**				
	Age (years), mean (SD)		-	-
	**Sex**			
		Male	-	-
		Female	-	-
	**Education level**		**-**	**-**
		Low	-	-
		Medium	-	-
		High	-	-
**BMI and behavioral factors**				
	BMI, kg/m^2^		-	-
	Fat intake, mean fat points/day		0.77 (0.62-0.95)	-
	Physical activity, mean minutes per day		0.98 (0.96-0.999)	-
**Motivational factors** ^b^				
	Intention for weight gain prevention, mean		-	-
	Perceived behavioral control for weight gain prevention, mean		-	-
**Self-regulation factors**				
	**Monitoring weight**		**-**	**-**
		Nonweekly		
		Weekly	-	-
	**Action planning,** ^c^ **mean**		**-**	**-**
		DI		
		PA	0.23 (0.05-0.97)	-
	Proactive coping skills for prevention of weight gain,^c^mean		-	0.28 (0.10-0.76)
	Restrained eating,^b^mean		**-**	2.45 (1.12-5.43)
	Weight locus of control,^c^mean		-	-

^a^Dashes indicate that the specific factor was not included in the final logistic model. In the last column, the last model with only statistically significant correlates is presented.

^b^Score range 1-5.

^c^Score range 1-4.

### Use of Planning Components

Server registrations showed that 140 (55.7%) of the participants chose to make a change in DI and 40 (15.9%) chose to make a change in PA; the other participants did not set a goal. Furthermore, 138 participants (54.9%) wrote an action plan for change in DI (Table 3), 111 (44.2%) of whom had a plan of good quality (clear description of situation and good plan). An action plan for an increase in PA was developed by 39 people (15.9%); 14 (5.6%) stated a plan of good quality. The most common reason for a plan to be considered of poor quality was that it gave an unclear description of the moment of change (eg, in the morning).

In total, 70 people (27.9%) indicated that they were expecting a risk situation for making a change in DI. A clear and helpful coping plan (clear description of high-risk situation and supportive coping plan) was stated by 50 (19.9%) participants, 12 participants (4.7%) were expecting a high-risk situation for a change in PA, and 6 of them (2.3%) wrote a clear and helpful coping strategy. Plans were deemed insufficient because they had either an unclear description of the situation or an unhelpful strategy.

**Table 3:** Frequency of use of the self-regulation tasks and quality of the generated plans among participants who visited the first module of the tailored intervention.

Target behavior	Self-regulation component	Yes^a^	Good quality
		n (%)	n (%)
Visit intervention	Visit first tailored module	251 (100)	**-**
**Dietary intake**			
	Chose a change in DI	140 (55.8)	**-**
	Set a goal for DI	138 (55.0)	111 (80.4)
	Described a coping plan for a change in DI	70 (27.9)	50 (71.4)
**Physical activity**			
	Chose a change in PA	40 (15.9)	**-**
	Set a goal for PA	40 (15.9)	14 (35.0)
	Described a coping plan for a change in PA	12 (4.8)	6 (50.0)

^a^The third column refers to the percentage of participants that have used certain parts of the tailored intervention. The second column refers to the percentage of participants who had stated a goal or plan of good quality (ie, obtained 2 points by the coding procedure). A dash indicates no data.

## Discussion

### Overview

In this study, we examined the reach and predictors of reach of an online computer-tailored weight gain prevention intervention for overweight adults. Initial use of the intervention was high (93.3%, 251/269), but only 26.4% (71/269) of the participants visited 3 modules and 14.9% (40/269) completed all 4 modules. Use of the first tailored intervention module was more likely among participants who had a lower fat intake, lower physical activity, and lower action planning for PA at baseline compared to those who never visited the intervention. Repeated use of the intervention was more likely among participants with higher levels of restrained eating and who had a lower score on proactive coping skills at baseline. Of those who used the tailored intervention, 55.8% (140/251) stated a goal for a change in DI and 15.9% (40/251) for a change in PA. Only 27.9% (70/251) made a coping plan for DI and 4.8% (12/251) for PA. Approximately half of the written goals and plans were of good quality.

### Website Reach and Characteristics of Users

Use of the first intervention module was high, 93.3% (251/269). However, only 15.2% (40/269) of all the participants finished the last (fourth) module. The modules required quite some effort because they were interactive and needed personal input for completion of questionnaires and formulation of action and coping plans. The sharpest decline in visits to the intervention was between the second and third visits. In the second visit, participants had to evaluate the success of their behavior change. It is possible that participants experienced this module as difficult, confrontational, or not supportive enough. The observed decline after the first module is comparable to what has been reported in evaluations of other online interventions [12-14,48], but is nevertheless worrisome. The email reminders sent every two weeks to (re)visit the intervention may have helped somewhat, but they were not sufficient to prevent the decline in follow-up visits. Other actions to increase revisiting might be helpful, for example, telephone calls or short initial face-to-face contact [10,48]. Short text messages may also be beneficial. They have been shown to improve the effects of a planning intervention on fat intake [11]. Including text messaging would also be an effective way to remind participants of their personal goals and plans, which is effective in increasing brisk walking [49].

This study showed that trait-like psychological factors, including body weight self-regulation skills and restrained eating, might influence online intervention use even more than behavioral or demographic factors. Restrained eaters were more likely to revisit the intervention. Perhaps this is because some characteristics related to restrained eating, such as high conscientiousness [50], may increase one’s intention to complete activities. Restrained eaters may also be extra motivated to find extra knowledge and strategies to control their dietary intake. Nonrestrained eaters may not be as motivated to complete the intervention. Perhaps they can be motivated in other ways, for example, by the promise of a self-introduced reward when finishing the program or achieving a goal.

Baseline weight-related proactive coping skills were negatively related to revisiting. This may indicate that those who could benefit most from the intervention (through learning planning and coping skills) were indeed more likely to use the intervention more often, whereas those who already had good coping skills may have felt that they were not sufficiently supported by the program. Therefore, the program may be adapted to fit the needs of those who already have good coping skills, but who have nevertheless not been successful in managing their weight.

Overall, these results indicate that self-regulation skills and traits that have previously been related to body weight have an influence on intervention-related behavior. As such, one must realize that choices that are made during intervention development (eg, theoretical framework and methods) may influence the motivation of certain groups to use the intervention. This is especially notable when looking at the differences between first and second time use; self-regulation factors had stronger relations with second time use (continuing use after module 1) than first time use.

Reach of the intervention was not associated with motivational factors. This contradicts the findings of other studies [12,13,51] that found that more motivated participants were more likely to revisit. An explanation of this difference may be that the self-regulation tools in this intervention were incorporated in a comprehensively tailored program and that the tailoring resulted in also attracting and committing participants with relatively lower levels of motivation to visit and revisit the intervention website. However, it may also be a consequence of the overall high motivation among participants, with little variance. Thus, the precise relation between motivation for behavior change and intervention use needs more exploration. Furthermore, future research could also include other individual predictors, such as disinhibition, taste, impulse control, and weight-related self-esteem.

Use and repeated use were also not related to sociodemographic characteristics. This may indicate that the program was equally appealing to people with higher and lower educational levels.

### Fidelity

More participants described a goal for change in DI compared to PA. This preference has been observed before in weight-related behavior studies [52-54]. Of the 269 participants, 66.9% (180) wrote down their behavior action plan, but the coping-planning component was used by only 30.5% (82/269). Similar figures have been reported in other online tailored interventions, for example by Spittaels and DeBourdeadhuiij [48], who found that only 3 of 6 people used the goal-planning component for improving PA. The goal-setting and action-planning components required active involvement of the participants (eg, self-reflection, thinking about a solution, writing it down). A lack of use of the goal-setting component is worrisome because the behavior change goal is the starting point for the rest of the intervention and a coping plan is beneficial for actual change. Therefore, it is very important to identify how the use of action- and coping-planning tools in online interventions can be promoted.

To our knowledge, this is one of the first studies to investigate fidelity in terms of looking at the quality of goals and plan from an open-ended entry approach [11]. In general, the quality of the participants’ action plans was higher than the quality of the coping plans. Coping planning is a more complex process than action planning and requires the identification of critical situations and then finding an appropriate and feasible solution [36].

The complexity of planning was also visible in another formative evaluation of a self-regulation intervention to promote PA among adolescents; it showed that participants often found it difficult to make detailed plans for a whole week [55]. Although the format in which participants plan certain activities in the first week is used by more interventions (eg, [56]), it may be too difficult to think this far ahead. Therefore, if the participant does not define a natural situation that is likely to be encountered (ie, situational cue), which was often the case in our study, the process of automated cue response cannot take place [38]. Exercises that do provide planning with a situational cue to promote the self-regulation of behavior or health have been applied in many other studies (eg, [57-59]), and have been found to be effective in an obese population [11]. However, most of these studies used a closed-ended format or a more intensive approach, such as 10 weekly group sessions [57]. For example, Lee and colleagues provide their participants with a tailored plan to be physically active for 30 minutes at least 5 days a week [60]. There is also evidence that action plans that are completed in the presence of a counsellor are more strongly related to behavior change [61], but the presence of a counselor may not necessarily lead to increased self-regulation of diet and PA [62]. However, this may also be related to quality differences between counsellors, which may be present even when they are trained, have practiced, and have received feedback [63].

Furthermore, even though computer tailoring mimics individual counseling to some extent, this interaction may not apply to action- and coping-planning components. The low use of the planning elements and quality of the goals and plans may indicate that this is a difficult task for participants or that it requires too much effort, at least in the way these planning components were incorporated into the present intervention. It is, therefore, very important to identify how the use and quality of action and coping plans in online interventions can be improved.

### Strengths and Limitations

One of this study's strengths is its use of objective information to assess the level of use of the website and the goal-setting and coping-planning tools. Additionally, we were able to link intervention use to personal characteristics, making it possible to describe characteristics of users and nonusers. Moreover, BMI was measured in an objective way. However, other correlates of intervention use were based on self-report, and it was not possible to compare the open-ended planning format to a closed-ended planning format. Finally, these results cannot be generalized to the whole population, because our participants were all overweight (BMI 25-30 kg/m^2^) and motivated to participate in this study.

### Conclusions

This trial showed that psychological factors such as self-regulation skills and action planning were associated with repeated use of an online, computer-tailored self-regulation intervention aimed at prevention of weight gain among overweight adults. For future research, including a wider variety of variables that may be related to intervention use can provide more insight into the factors that are related to intervention use. Reach of the intervention was not optimal, with relatively few participants visiting all the intervention modules. The use of the action- and coping-planning components of the intervention was even lower and the quality of the generated plans was disappointing, especially for the coping plans. It is important to identify how overall reach of the intervention can be improved, as well as use and quality of action-planning and coping-planning components.

## Multimedia Appendix 1

Screenshots from the GRIPP intervention, showing the steps of the tailored intervention.

## Multimedia Appendix 2

Description of measurements.

## Multimedia Appendix 3

CONSORT-EHEALTH checklist V1.6.2 [64].

## Abbreviations

DI: dietary intake
PA: physical activity
