# Supplementary material for: Intervention Use and Action Planning in a Web-Based Computer-Tailored Weight Management Program for Overweight Adults: Randomized Controlled Trial
Source: JMIR Res Protoc. 2014 Jul 23;3(3):e31. doi: 10.2196/resprot.2599 (PMC4129126; doi:10.2196/resprot.2599)
Supplement: Supplementary file 1 [file resprot_v3i2e31_app1.pdf]

Information op website www.gripprogramma.nl:

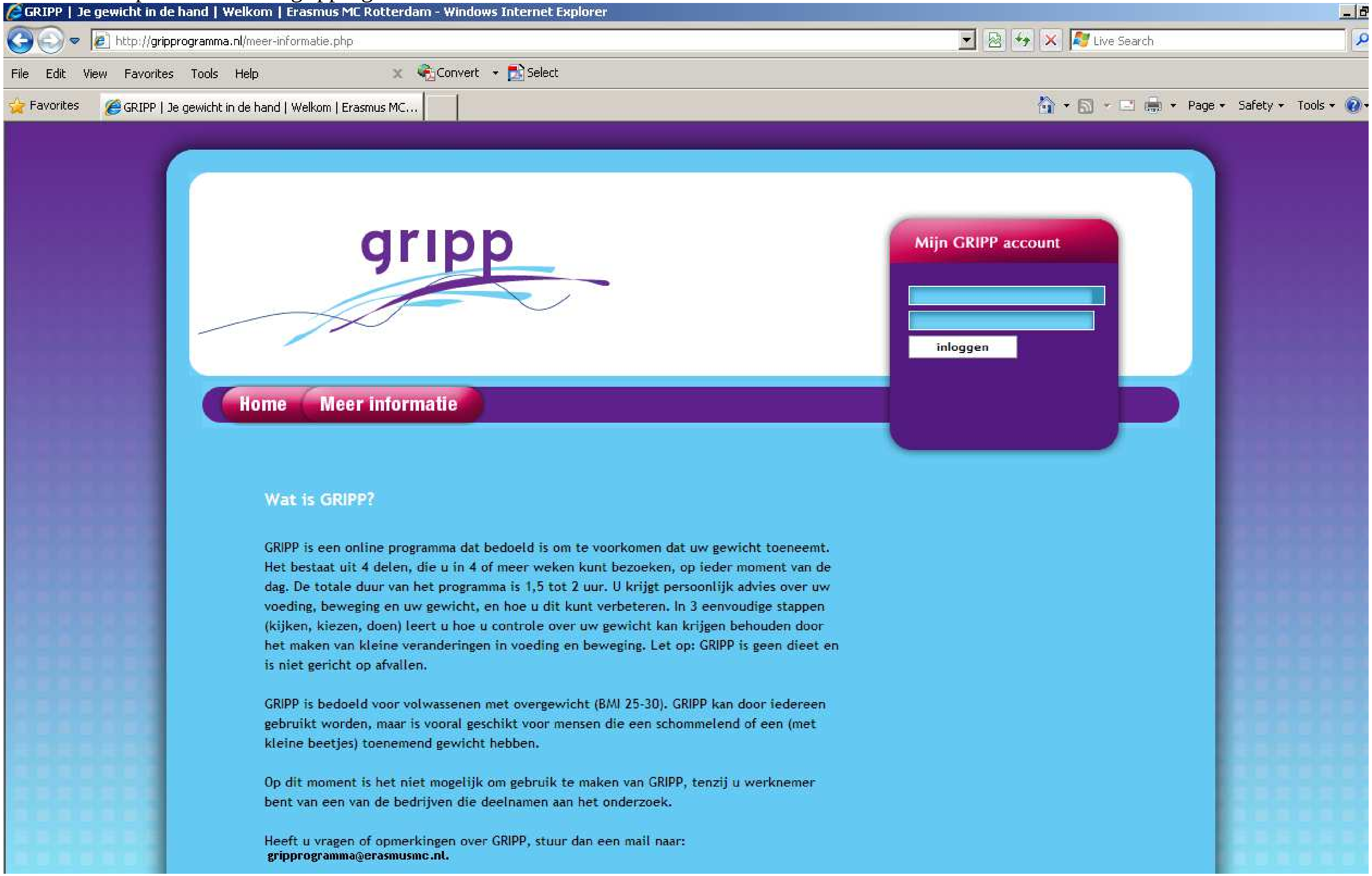

Information after logging in:

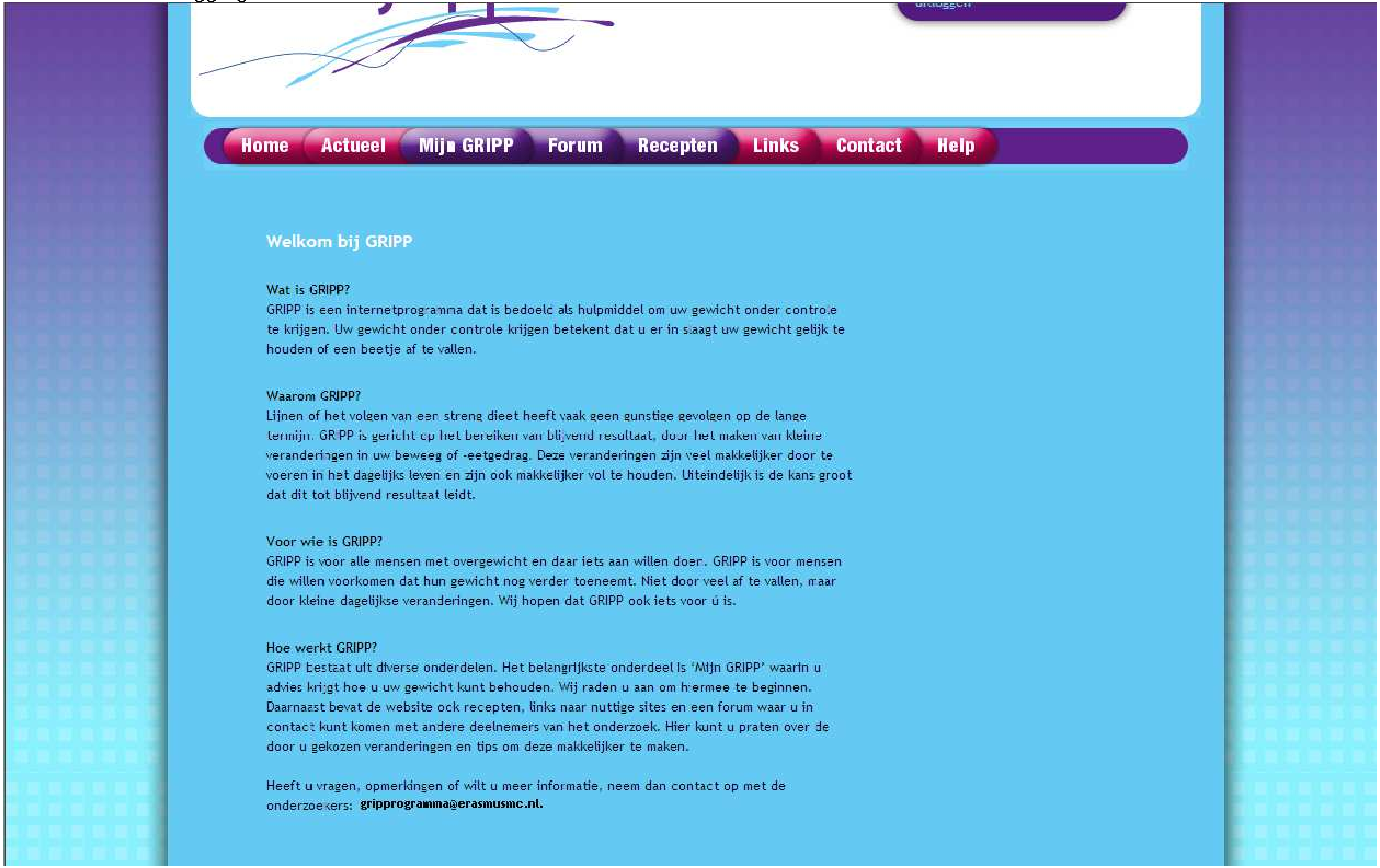

Home Actueel Mijn GRIPP Forum Recepten Links Contact Help

Welkom bij GRIPP

Wat is GRIPP?
GRIPP is een internetprogramma dat is bedoeld als hulpmiddel om uw gewicht onder controle te krijgen. Uw gewicht onder controle krijgen betekent dat u er in slaagt uw gewicht gelijk te houden of een beetje af te vallen.

Waarom GRIPP?
Lijnen of het volgen van een streng dieet heeft vaak geen gunstige gevolgen op de lange termijn. GRIPP is gericht op het bereiken van blijvend resultaat, door het maken van kleine veranderingen in uw beweeg of -eetgedrag. Deze veranderingen zijn veel makkelijker door te voeren in het dagelijks leven en zijn ook makkelijker vol te houden. Uiteindelijk is de kans groot dat dit tot blijvend resultaat leidt.

Voor wie is GRIPP?
GRIPP is voor alle mensen met overgewicht en daar iets aan willen doen. GRIPP is voor mensen die willen voorkomen dat hun gewicht nog verder toeneemt. Niet door veel af te vallen, maar door kleine dagelijkse veranderingen. Wij hopen dat GRIPP ook iets voor ú is.

Hoe werkt GRIPP?
GRIPP bestaat uit diverse onderdelen. Het belangrijkste onderdeel is 'Mijn GRIPP' waarin u advies krijgt hoe u uw gewicht kunt behouden. Wij raden u aan om hiermee te beginnen. Daarnaast bevat de website ook recepten, links naar nuttige sites en een forum waar u in contact kunt komen met andere deelnemers van het onderzoek. Hier kunt u praten over de door u gekozen veranderingen en tips om deze makkelijker te maken.

Heeft u vragen, opmerkingen of wilt u meer informatie, neem dan contact op met de onderzoekers: **gripprogramma@erasmusmc.nl.**

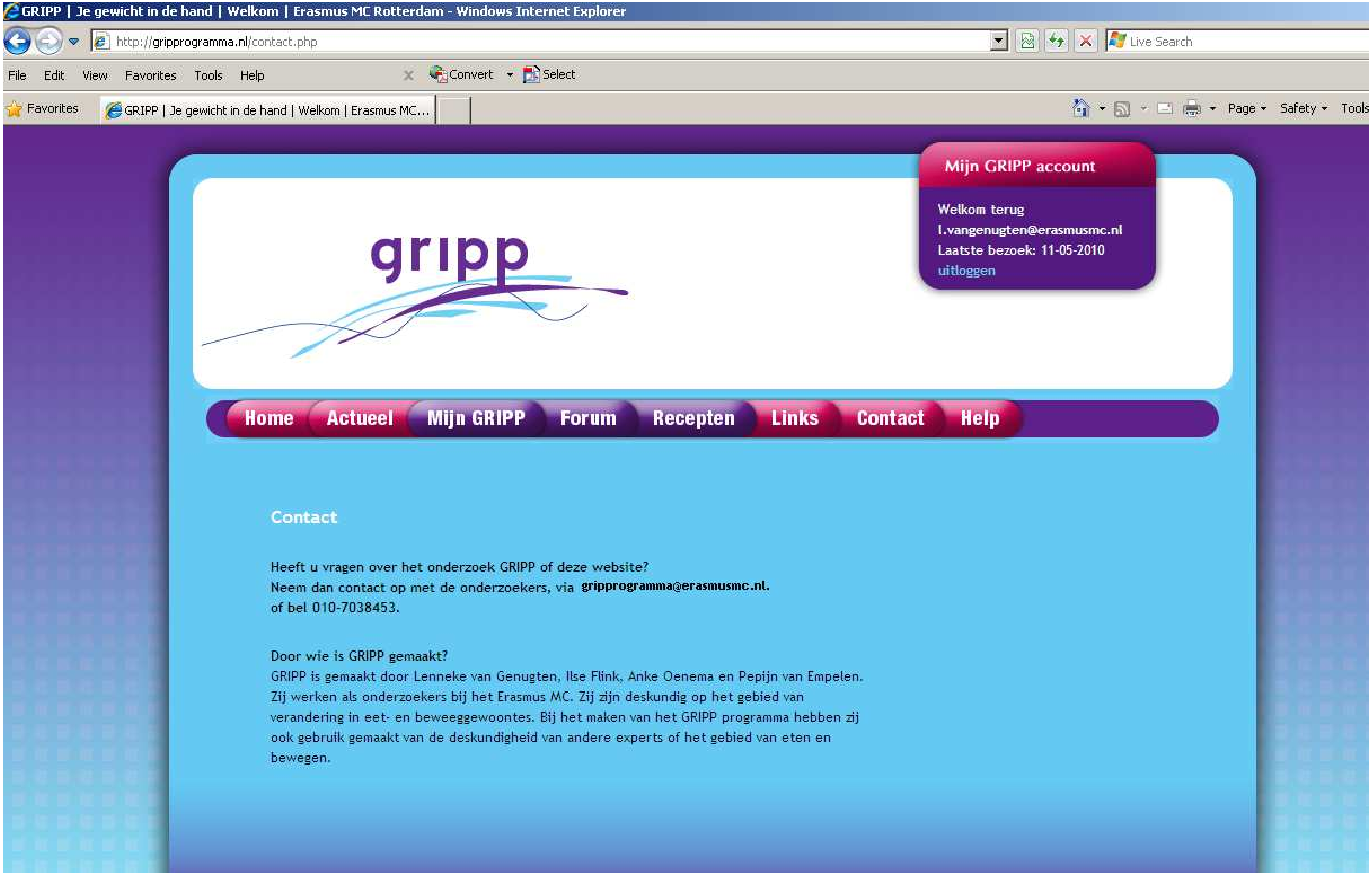
GRIPP | Je gewicht in de hand | Welkom | Erasmus MC Rotterdam - Windows Internet Explorer
http://gripprogramma.nl/contact.php
File Edit View Favorites Tools Help
Convert Select
Favorites
GRIPP | Je gewicht in de hand | Welkom | Erasmus MC...
Page Safety Tools
gripp
Mijn GRIPP account
Welkom terug
l.vangenugten@erasmusmc.nl
Laatste bezoek: 11-05-2010
uitloggen
Home Actueel Mijn GRIPP Forum Recepten Links Contact Help
Contact
Heeft u vragen over het onderzoek GRIPP of deze website?
Neem dan contact op met de onderzoekers, via gripprogramma@erasmusmc.nl.
of bel 010-7038453.
Door wie is GRIPP gemaakt?
GRIPP is gemaakt door Lenneke van Genugten, Ilse Flink, Anke Oenema en Pepijn van Empelen.
Zij werken als onderzoekers bij het Erasmus MC. Zij zijn deskundig op het gebied van
verandering in eet- en beweeggewoontes. Bij het maken van het GRIPP programma hebben zij
ook gebruik gemaakt van de deskundigheid van andere experts of het gebied van eten en
bewegen.

Home Actueel Mijn GRIPP Forum Recepten Links Contact Help

Help

De meest gestelde vragen over GRIPProgramma.nl:

V: Er gebeurt niets als ik op 'Mijn GRIP' of 'Forum' klik.
A: Beide toepassingen openen zich in een nieuw venster. Klik nogmaals op de knop, maar houdt hierbij de 'control' toets ingedrukt. Of klik op de link die op de pagina verschijnt.

V: Ik krijg een leeg scherm als ik op mij GRIPP klik.
A: Het duurt vrij lang voordat het programma laadt. Houd alstublieft geduld. Gebeurt er na enkele minuten nog niets? Sluit het venster en probeer het nogmaals.

V: Ik wil inloggen, maar het lukt niet.
A: Controleer uw wachtwoord nogmaals. Heeft u het goed ingetypt? Let ook op de hoofdletters en andere tekens, het is belangrijk dat u deze juist overneemt.

V: Mijn vrouw/kind/kennis/buurman wil ook graag mee doen. Kan ik mijn inlognaam geven?
A: Nee, uw inlognaam is strikt persoonlijk en kan niet door meerdere mensen gebruikt worden. Hierdoor zal het programmam voor u niet meer kloppen. De geïnteresseerde persoon kan hiervoor contact opnemen met gripprogramma@erasmusmc.nl

V: Ik heb geen tijd om de site meerdere malen te bezoeken, kan het ook in 1 keer?
A: Nee, voor de werking van het programma is het erg belangrijk dat er minstens een week tussen de bezoeken in zit.

V: Ik heb deze week geen tijd om het programma te bezoeken, wat nu?
A: Het is geen probleem als er meer dan een week tussen 2 bezoeken zit. Probeer dit echter niet meer dan 2 weken te laten worden.

Staat uw vraag er niet bij? Stuur dan een mail naar **gripprogramma@erasmusmc.nl.**

The user forum

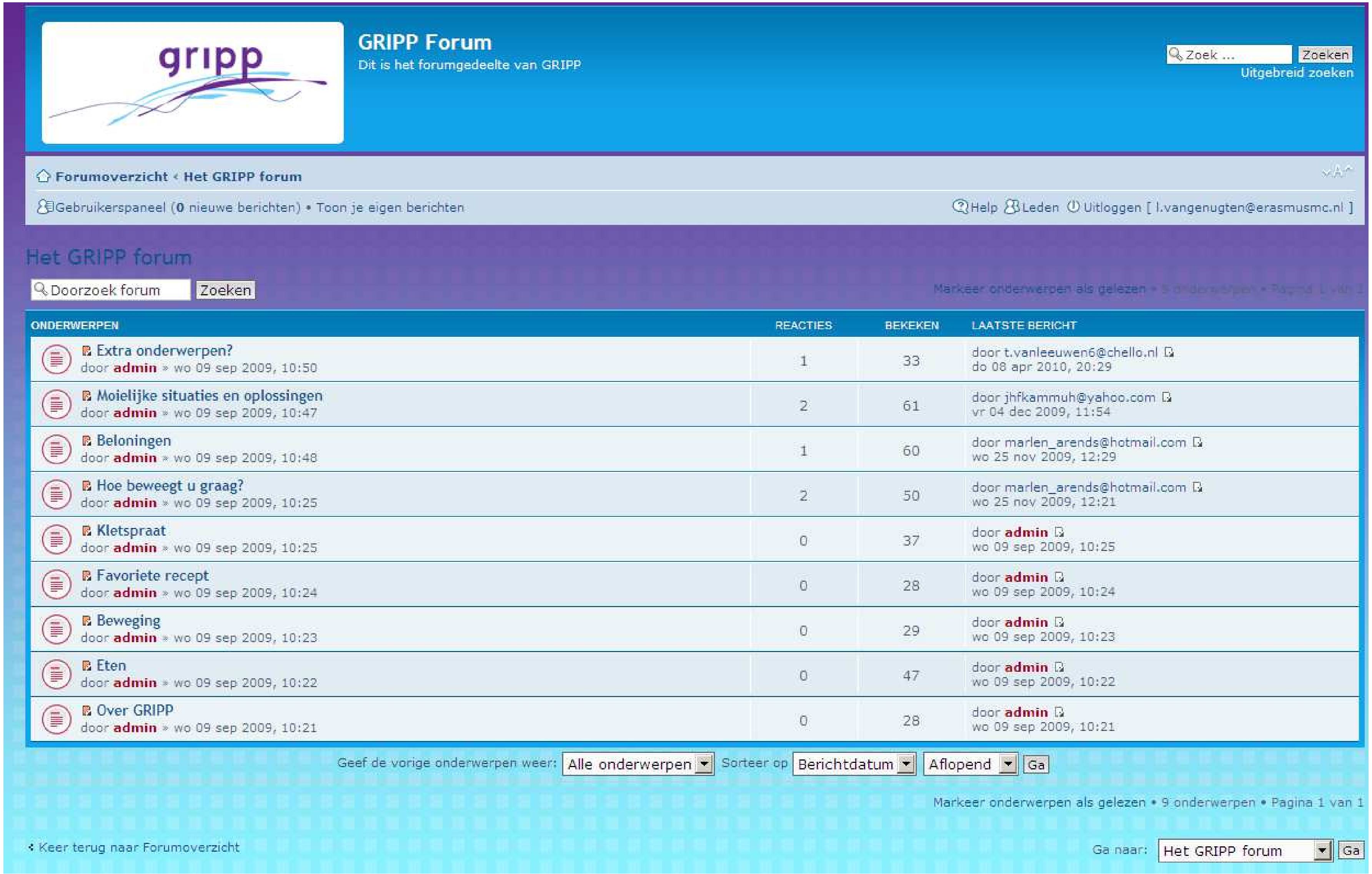

The recipe database:

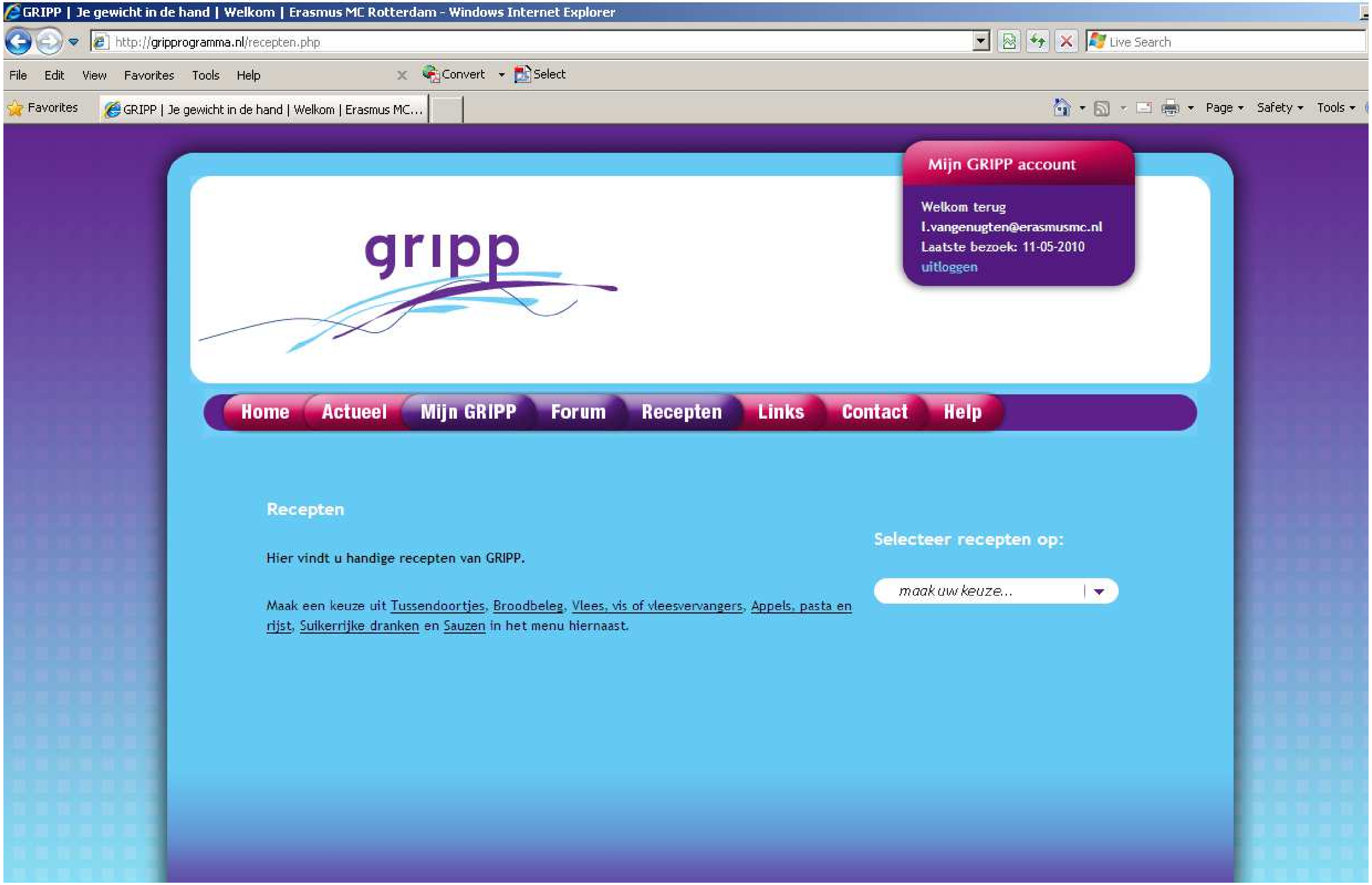

Links to useful other sites

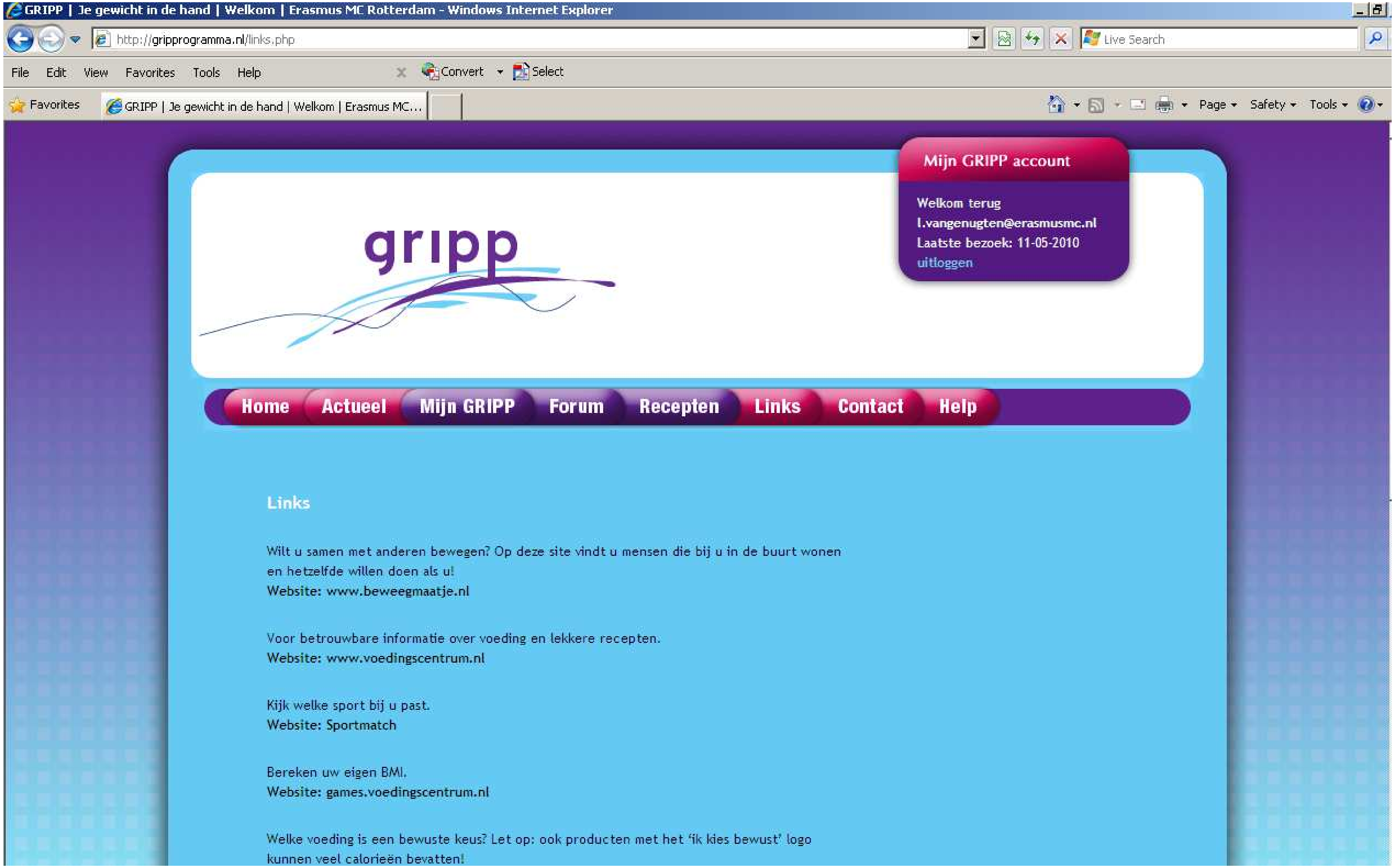

Tha tailored part. Module 1.

Welkom bij GRIPP!

Hoe werkt GRIPP?
Tijdens uw vier bezoeken aan GRIPP doorloopt u de volgende onderdelen:

Bezoek 1: Kijken naar uw leefstijl en een kleine verandering kiezen en plannen
Bezoek 2: Kijken hoe de kleine verandering is gegaan
Bezoek 3: Uw kleine verandering aanpassen zodat u hem vol blijft houden
Bezoek 4: Afsluiting en voorbereiding op de toekomst

Om alle stappen goed te kunnen doorlopen, krijgt u eerst wat persoonlijke vragen, onder meer over uw gewicht. Het is belangrijk dat u deze en alle andere vragen in het programma zo eerlijk mogelijk invult. Op die manier kunnen wij u een zo persoonlijk mogelijk advies geven.

Instructies
Voordat u gaat beginnen nog kort wat instructies over hoe het programma werkt.

- Om naar een volgende pagina te gaan kunt u op de knop volgende klikken. Deze knop staat onderaan de pagina. Als u op een andere knop of kopje moet klikken om verder te gaan dan krijgt u hier instructies over.
- Wilt u terug naar een pagina, dan kunt u op de knop vorige klikken. Ook deze staat onderaan de pagina.
- Als u begint aan een nieuw onderdeel of halverwege bent ziet u onderaan de pagina een roze statusbalk staan. Deze geeft aan hoe ver u in het programma bent. Deze balk staat onderaan de pagina.
- U kunt het programma op elk willekeurig moment verlaten. Als u opnieuw inlogt, komt u vanzelf terecht bij de vraag die u als laatste hebt ingevuld.

Veel plezier met GRIPP!

Wat is uw voornaam?

Linda

Bent u man of vrouw?

- ( ) Man
- (•) Vrouw

Wat is uw geboortedatum?
Geeft dit weer als dd-mm-jjjj, bijvoorbeeld 13-09-1963.

18-10-1983

Bij de volgende twee vragen kunt u, uw antwoord alleen in cijfers weergeven. Gebruik bij deze vragen <u>geen</u> spaties.

Wat is uw leeftijd op dit moment?

26

Wat is uw lengte (in centimeters)?

174

Wat is uw gewicht in kilogrammen? (de laatste keer dat u zich op de hieronder beschreven manier gewogen hebt)

85

TIP: Om uw gewicht nauwkeurig te bepalen kunt u gebruik maken van een weegschaal. U kunt uzelf dan het beste op een vast tijdstip wegen (bijvoorbeeld s'ochtends voor het ontbijt) en elke keer dezelfde weegschaal gebruiken. Trek dan in ieder geval uw schoenen uit (bij voorkeur houdt u alleen uw onderkleding

Welke situatie omschrijft uw gewichtsverloop van de afgelopen vijf jaar tot nu het best?

- ○ Mijn gewicht is afgenomen
- ○ Mijn gewicht is gelijk gebleven
- ○ Mijn gewicht heeft geschommeld
- ◉ Mijn gewicht is toegenomen

Heeft u in de afgelopen vijf jaar geprobeerd af te vallen door te lijnen?

- ○ Ja
- ◉ Nee

Stap 1: Beginnen met GRIPP

Voordat u begint met uw eigen gewicht onder controle te krijgen, is het goed om wat meer te weten over gewichtscontrole. Wat ís gewichtscontrole eigenlijk, hoe kun je het aanpakken en welke voor- en nadelen heeft het voor u?

Het eerste onderdeel van GRIPP gaat hier op in door de volgende onderwerpen:

- de energiebalans
- waarom is het belangrijk om uw gewicht onder controle te krijgen
- hoe kun je gewichtscontrole bereiken
- wat kan gewichtscontrole voor u betekenen
- uw eigen afweging van voor- en nadelen van gewichtscontrole

Om te kijken of gewichtscontrole iets voor u is, gaan we eerst iets verder in op de energiebalans.

Klik op de link hieronder om verder te gaan.

Klik dan hier om te beginnen

Weten wat energiebalans is

De energiebalans: balans tussen wat u eet en drinkt en hoeveel u beweegt

Om GRIP op uw gewicht te krijgen is het belangrijk om iets te weten van de energiebalans. GRIP op gewicht betekent eigenlijk het krijgen en houden van een goede balans tussen wat u eet en drinkt en hoeveel u beweegt.

Hoe werkt de energiebalans?
U kunt de energiebalans vergelijken met een ouderwetse weegschaal. Door te eten en te drinken krijgt u een bepaalde hoeveelheid calorieën (ook wel energie genoemd) binnen. Door te bewegen (bijvoorbeeld wandelen, fietsen, sporten) verbruikt u calorieën of energie. Als de hoeveelheid energie die u binnenkrijgt door wat u eet en drinkt gelijk is aan de energie die u verbruikt door te bewegen, is de weegschaal in balans. Uw gewicht blijft dan gelijk. U kunt dat hieronder in het plaatje zien.

Uit balans
Als uw gewicht toeneemt (u wordt dus zwaarder) betekent dit dat uw energiebalans uit balans is. U krijgt dan meer energie binnen door het eten en drinken dan u verbruikt door te bewegen. Een klein verschil tussen energie inname en verbruik kan op den duur leiden tot een grote toename in gewicht.

Weer in balans
Een energiebalans die uit balans is, kan ook weer in balans komen. Om weer in balans te komen zijn niet heel grote veranderingen nodig. Kleine, maar blijvende veranderingen in wat u eet en drinkt en/of hoeveel u beweegt kunnen al genoeg zijn.

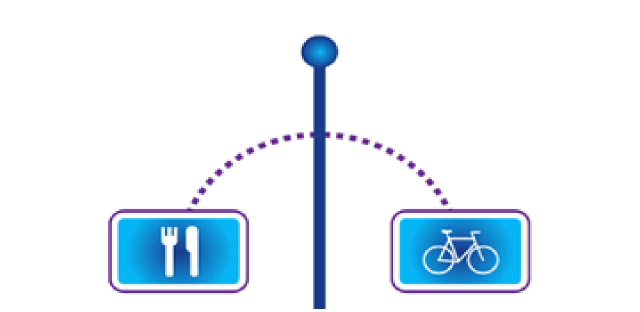

Weten waarom het belangrijk is uw gewicht onder controle te krijgen

Voorkomen van gewichtstoename

GRIP krijgen en houden op uw gewicht is belangrijk om te voorkomen dat uw gewicht verder toeneemt. Als uw energiebalans langdurig uit evenwicht is (u krijgt dus meer energie binnen dan u verbruikt) wordt u steeds een beetje zwaarder. Aanvankelijk lijkt dat misschien niet zo erg. Maar als uw gewicht maar blijft toenemen dan kan dat uiteindelijk leiden tot zwaar overgewicht. Zwaar overgewicht wordt ook wel obesitas genoemd. Zwaar overgewicht kan nadelige gevolgen hebben voor uw gezondheid.

In het onderstaande plaatje kunt u zien welke nadelige gevolgen dat zijn. Als u op de verschillende lichaamsdelen klikt dan kunt u zien wat de gevolgen zijn voor dat lichaamsdeel. Uit onderzoek blijkt dat het heel moeilijk is om blijvend af te vallen als er eenmaal sprake is van zwaar overgewicht. Daarom is het belangrijk om zwaar overgewicht te voorkomen en nú GRIP te krijgen op uw gewicht.

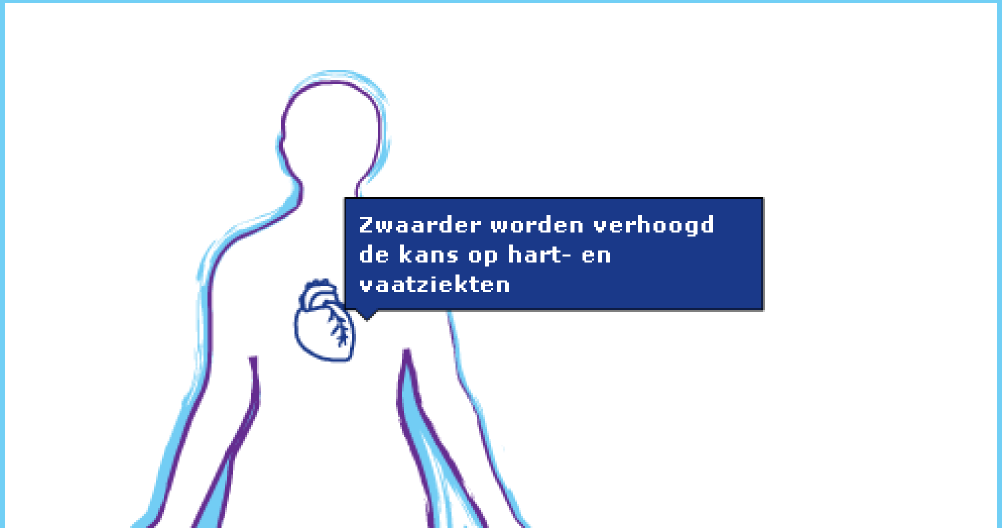

Kijken naar uw gewichtsverloop

Steeds een beetje zwaarder…

U gaf eerder aan dat uw gewicht is toegenomen de afgelopen 5 jaar. Dit kunt u zien in deze grafiek.

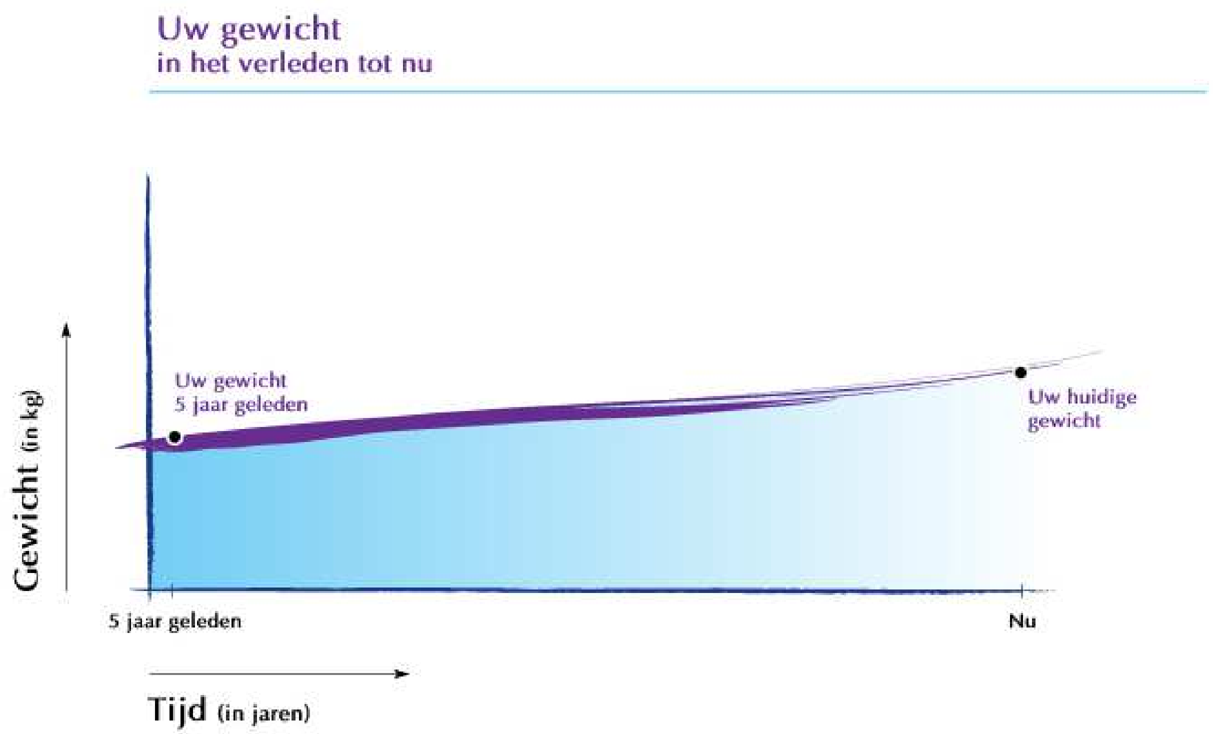

Kleine veranderingen om GRIP op uw gewicht te krijgen

Wat is er nodig om GRIP op uw gewicht te krijgen?

U kunt GRIP op uw gewicht krijgen door kleine veranderingen te maken in wat u eet en drinkt en/of hoeveel u beweegt.

Om GRIP op uw gewicht te krijgen is het dus niet nodig om een dieet te gaan volgen of intensief te gaan sporten. Meestal werkt dat juist niet. Diëten vergen zulke grote aanpassingen in uw dagelijks leven, dat die op de lange duur vaak niet vol te houden zijn. Kleine veranderingen in wat u eet en hoeveel u beweegt zijn goed in te passen in het dagelijks leven en daarom beter vol te houden.

Het is wel belangrijk om te kiezen voor kleine veranderingen die bij ú passen en die u kunt inpassen in uw dagelijks leven. Het GRIP programma is een hulpmiddel om daar goede keuzes in te maken.

Om u vast een idee te geven van mogelijke veranderingen, staan hieronder een paar voorbeelden van veranderingen die anderen hebben gekozen.

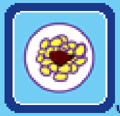
Jannet (43, verpleegkundige, 2 kinderen) koos ervoor om bij de avondmaaltijd kleinere porties te nemen. In plaats van nog een tweede keer op te scheppen, schept ze nu maar 1 keer op.

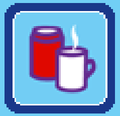
Linda (40, secretaresse) koos ervoor om in plaats van gewone cola, cola light te gaan drinken.

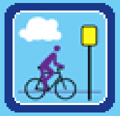
Pascalle (39, verpleegkundige, 3 kinderen) koos ervoor om twee keer per week te gaan roeien, lekker ontspannen in de buitenlucht.

U gaf aan dat u over 5 jaar graag veel wilt afvallen. Waarom wilt u dit graag? Kies de reden die u het belangrijkst vindt.

- (•) Daardoor word ik gezonder
- ( ) Ik wil slanker worden
- ( ) Dat ziet er mooier uit
- ( ) Ik kan nu geen leuke kleren aan
- ( ) Ik moet afvallen van een arts of andere gezondheidsspecialist
- ( ) Dan kan ik trots zijn op mezelf
- ( ) Ik wil weer net zoveel wegen als toen ik jong was
- ( ) Ik wil fitter worden
- ( ) Ik zit dan lekkerder in mijn vel
- ( ) Anders

Afvallen en gezondheid

U gaf aan dat u de komende vijf jaar flink wilt afvallen omdat dit goed zou zijn voor uw gezondheid. Hoewel dit inderdaad zo is, kan een aanzienlijk gewichtsverlies ook negatieve invloed hebben op uw gezondheid. Dit komt allemaal door het "jojo" effect waar u eerder over heeft gelezen. Gezondheidsdeskundigen hebben berekend dat het erg ongezond is om eerst veel af te vallen en daarna weer aan te komen. Zij adviseren daarom om het gewicht stabiel te houden door een gezond eet- en beweegpatroon aan te nemen. Als dit lukt voor een lange periode, kunt u daarna gaan proberen om langzaam enkele kilo's te verliezen. Zo kunt u uw gezondheid verbeteren zonder te diëten!

De voor- en nadelen van gewichtscontrole

Gewichtscontrole is: *Zorgen dat je net zoveel energie binnenkrijgt als dat je verbruikt waardoor je energiebalans in balans is.*

Om dit te bereiken kunt u één of meerdere kleine veranderingen maken in uw leefstijl. Dat kan dan een verandering zijn in wat u eet of in hoeveel u beweegt.

Gewichtscontrole heeft voor- en nadelen. Maar ook uw gedrag niet veranderen heeft gevolgen. Wat zijn voor u de belangrijkste voor- en nadelen van gewichtscontrole? U kunt ze hieronder en op de volgende pagina op een rijtje zetten.

Wat zijn voor u mogelijke nadelen van gewichtscontrole?
Nadeel 1:

```
Het is misschien wel moeilijk.
```

Nadeel 2:

```
Ik wil liever afvallen.
```

Andere mensen noemden deze nadelen:

- Dan moet ik m'n gedrag veranderen
- Het kan tijd kosten
- [illegible]

De voor- en nadelen van gewichtscontrole

Wat zijn voor u de belangrijkste twee **voordelen** van gewichtscontrole?

Probeer deze voordelen op te schrijven als een persoonlijke motivatie.

Voordeel 1:

Dan word ik niet zwaarder

Voordeel 2:

Geen

Andere mensen noemden deze voordelen

- Ik voel me fitter als ik mijn gewicht onder controle heb.
- Ik moet een kleine verandering maken en blijven volhouden.
- Ik hoef niet elk jaar weer nieuwe kleding te kopen als ik mijn gewicht onder controle heb.
- Dan baal ik niet van mezelf als ik toch aankom.
- Voorkomen is beter dan genezen

U hebt net aangegeven dat gewichtscontrole voor u de volgende voor- en nadelen heeft:

**Voordelen van gewichtscontrole**	**Nadelen van gewichtscontrole**
Dan word ik niet zwaarder	*Het is misschien wel moeilijk.*
Geen	*Ik wil liever afvallen.*

Stilstaan bij de voor- en nadelen

Als u naar de voor- en nadelen kijkt, wat weegt er dan het zwaarst? Welke voor- of nadelen zijn het belangrijkst? Zijn de nadelen echte nadelen? Bedenk ook dat sommige dingen die nu een nadeel lijken, minder belangrijk worden als u eenmaal succesvol bezig bent met gewichstcontrole .

Nu u heeft nagedacht over de voor- en nadelen, denkt u dan:

1. Gewichtscontrole iets is wat ik wil proberen.

- (●) Eens
- ( ) Oneens

2. Gewichtscontrole iets is wat ik kan

- ( ) Eens
- (●) Oneens

Op gewicht blijven

U gaf aan dat u nog niet zeker weet of het u gaat lukken om op gewicht te blijven. Dat is begrijpelijk, want het kan best moeilijk zijn. Toch zou het u - met de juiste ondersteuning en goede tips en adviezen - kunnen lukken. GRIPP is daarvoor een hulpmiddel.

Maar, misschien helpt het ook om te kijken naar wat andere mensen aan het programma hebben gehad:

"Ik kwam erachter dat ik mijn eetpatroon op een hele eenvoudige manier en zonder dieet kan aanpassen"
"Je kan dingen anders doen en er toch lol in blijven houden!"
"Ik vind het fijn dat ik met kleine veranderingen toch iets kan verbeteren en zelf kan bepalen wat ik dan kies"

Als u verder wilt gaan met GRIPP klik dan op 'volgende'. U kunt dan kijken naar uw eet- en beweeggewoontes.

Participant scan choose whether they’d like to receive feedback on their dietary intake, physical activity or both.

Kijken naar wat u eet en hoeveel u beweegt

In het volgende onderdeel van GRIPP kunt u nagaan wat u normaal gesproken eet en drinkt en hoeveel u beweegt op een dag. Dit kunt u doen door vragen over eten en bewegen te beantwoorden. Op basis van uw antwoorden krijgt u dan in een persoonlijk advies een overzicht van de kleine veranderingen die ú zou kunnen maken om GRIP op uw gewicht te krijgen.

U kiest vervolgens **zelf** welke kleine verandering **u** zou willen maken.
U kunt hieronder aangeven waar u eerst naar wilt kijken, naar eten, of naar bewegen.

- (•) Ik wil kijken naar wat ik eet en drink
- ( ) Ik wil kijken naar hoeveel ik beweeg
- ( ) Ik weet het nog niet

Kijken naar wat u eet

U kunt nu de vragen invullen over wat u gewoonlijk eet. Er wordt vooral gevraagd naar voedingsmiddelen die in een gemiddelde Nederlandse voeding het meest bijdragen aan de energie-inname, oftewel de hoeveelheid calorieën in de voeding. Na het beantwoorden van de laatste vraag, krijgt u een persoonlijk advies met daarin suggesties voor kleine veranderingen die u zou kunnen maken.

- Denk bij het beantwoorden van de vragen aan wat u de afgelopen maand meestal at of dronk
- Er zijn geen goede of foute antwoorden
- Kies het antwoord dat het beste bij u past

ZUIVEL

	Nooit of minder dan 1 dag per week	1 dag per week	2 dagen per week	3 dagen per week	4 dagen per week	5 dagen per week	6 dagen per week	7 dagen per week
Hoeveel dagen per week drinkt u gewoonlijk karnemelk of ongezoete zuiveldranken zoals optimel?	○	○	○	○	○	○	○	◉
Hoeveel dagen per week drinkt u gewoonlijk melk?	◉	○	○	○	○	○	○	○
Hoeveel dagen per week drinkt u gewoonlijk gezoete zuiveldranken, zoals Yogho-Yogho, Biomild, Fristi, Milk&Fruit, Smooties, Vifit e.d.?	◉	○	○	○	○	○	○	○

Hoeveel bekers karnemelk neemt u dan meestal per dag?

- (•) 1 per dag
- ( ) 2 per dag
- ( ) 3 per dag
- ( ) 4 per dag
- ( ) 5 of meer per dag

Welke soort vleeswaren doet u meestal op uw brood, cracker of beschuit?

- ( ) bacon, bloedworst, boterhamworst, gebraden gehakt, cervelaatworst, cornedbeef, leverworst, paté, palingworst, ontbijtspek, salami, smeerleverworst, etc.
- ( ) beenham, rauwe hamsoorten zoals Serrano ham, Schwartzwalder Schinken, Kempische, Ardenner ham, Coburger ham
- ( ) rookvlees, rosbief, magere rollade, fricandeau, schouderham, achterham, kipfilet, casselerib, etc.
- ( ) wisselend

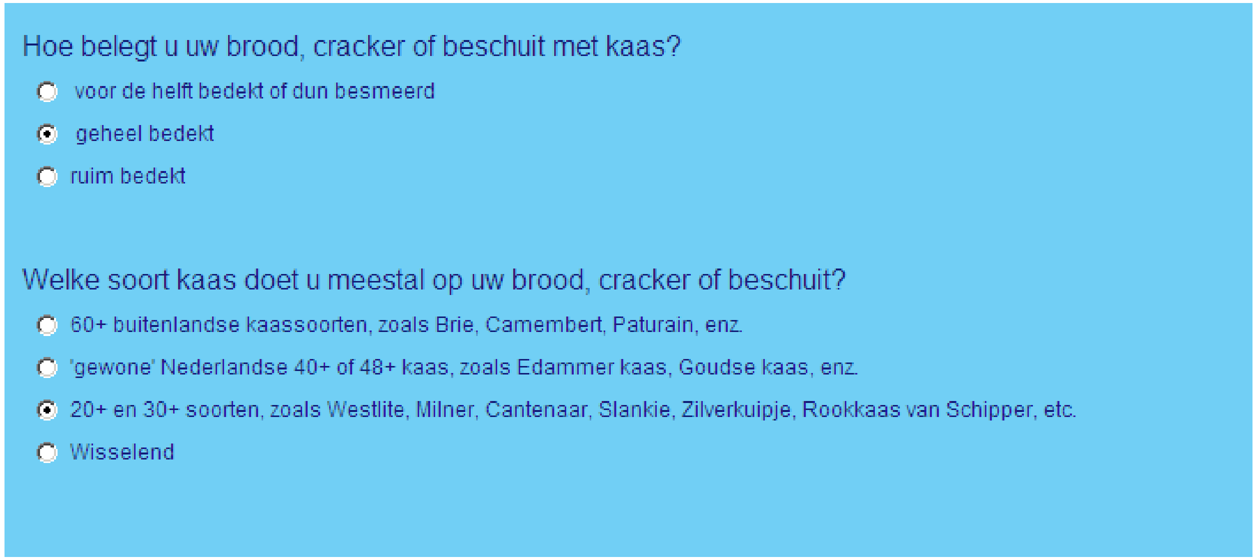
Hoe belegt u uw brood, cracker of beschuit met kaas?

- ( ) voor de helft bedekt of dun besmeerd
- (•) geheel bedekt
- ( ) ruim bedekt

Welke soort kaas doet u meestal op uw brood, cracker of beschuit?

- ( ) 60+ buitenlandse kaassoorten, zoals Brie, Camembert, Paturain, enz.
- ( ) 'gewone' Nederlandse 40+ of 48+ kaas, zoals Edammer kaas, Goudse kaas, enz.
- (•) 20+ en 30+ soorten, zoals Westlite, Milner, Cantenaar, Slankie, Zilverkuipje, Rookkaas van Schipper, etc.
- ( ) Wisselend

De questionnaire measures the following aspects: (days/week, intake per day en fat/sugar content (normal, fat free, sugar free, etc):

- Fruit and vegetables
- Sandwich fillings
- Sweet drinks (juice, soda, coffee, tea)
- alcohol
- sauces and gravy
- meat, fish, cheese and vegetarian replacements
- potatoes, pasta and rice
- snacks: sweet (chocolate, cookies, etc) salty (cheese, spring rolls, french fries), hot and cold.

AARDAPPELS, PASTA EN RIJST

	Nooit of minder dan 1 dag per week	1 dag per week	2 dagen per week	3 dagen per week	4 dagen per week	5 dagen per week	6 dagen per week	7 dagen per week
Hoe vaak gebruikt u aardappels bij de warme maaltijd?	○	○	○	○	○	○	○	○
Hoe vaak gebruikt u pasta bij de warme maaltijd?	○	○	○	○	○	○	○	○
Hoe vaak gebruikt u rijst bij de warme maaltijd?	○	○	○	○	○	○	○	○

Based on their answers, users receive personal feedback.

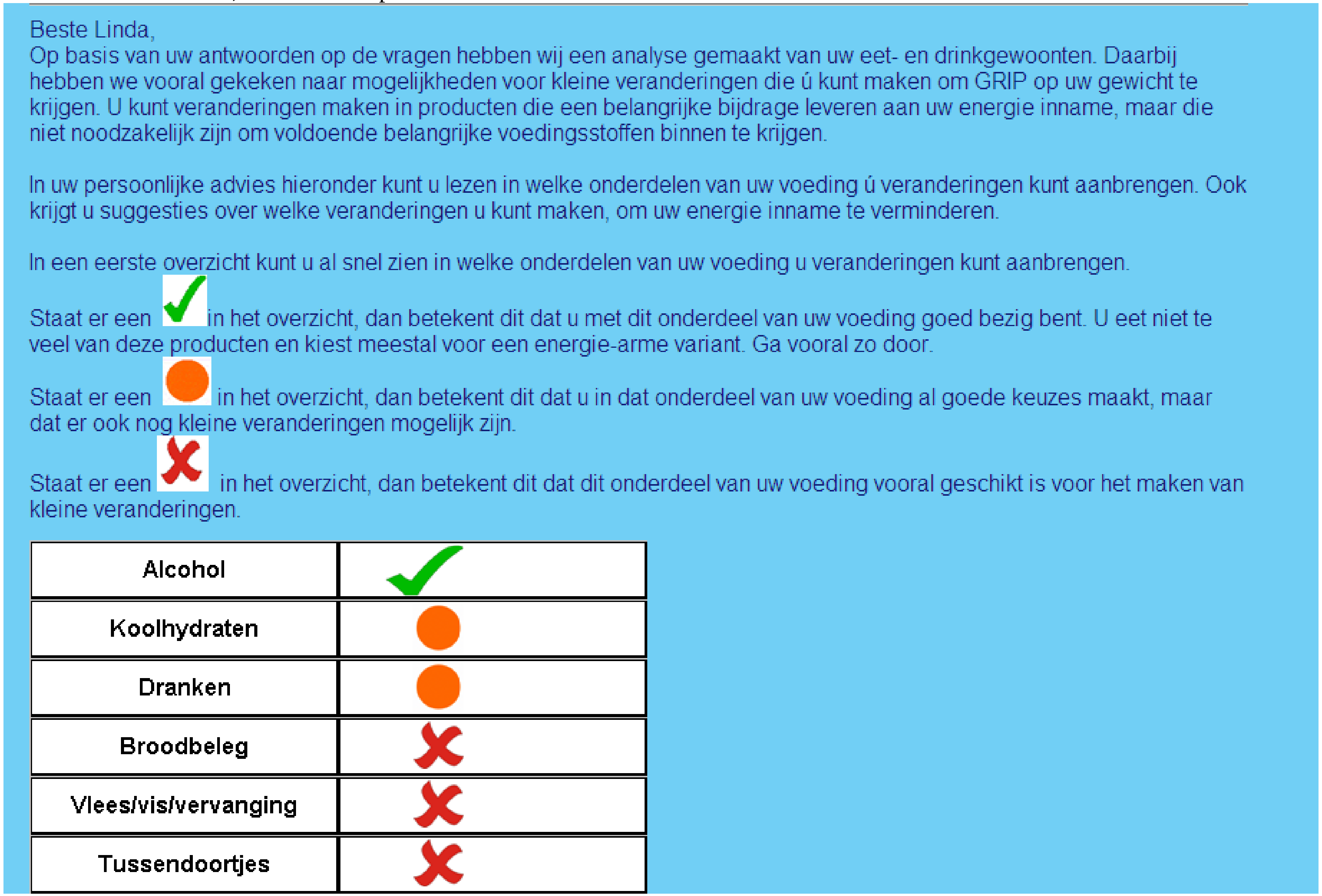

Beste Linda,
Op basis van uw antwoorden op de vragen hebben wij een analyse gemaakt van uw eet- en drinkgewoonten. Daarbij hebben we vooral gekeken naar mogelijkheden voor kleine veranderingen die ú kunt maken om GRIP op uw gewicht te krijgen. U kunt veranderingen maken in producten die een belangrijke bijdrage leveren aan uw energie inname, maar die niet noodzakelijk zijn om voldoende belangrijke voedingsstoffen binnen te krijgen.

In uw persoonlijke advies hieronder kunt u lezen in welke onderdelen van uw voeding ú veranderingen kunt aanbrengen. Ook krijgt u suggesties over welke veranderingen u kunt maken, om uw energie inname te verminderen.

In een eerste overzicht kunt u al snel zien in welke onderdelen van uw voeding u veranderingen kunt aanbrengen.

Staat er een ✓ in het overzicht, dan betekent dit dat u met dit onderdeel van uw voeding goed bezig bent. U eet niet te veel van deze producten en kiest meestal voor een energie-arme variant. Ga vooral zo door.

Staat er een ● in het overzicht, dan betekent dit dat u in dat onderdeel van uw voeding al goede keuzes maakt, maar dat er ook nog kleine veranderingen mogelijk zijn.

Staat er een ✗ in het overzicht, dan betekent dit dat dit onderdeel van uw voeding vooral geschikt is voor het maken van kleine veranderingen.

Alcohol	✓
Koolhydraten	●
Dranken	●
Broodbeleg	✗
Vlees/vis/vervanging	✗
Tussendoortjes	✗

They receive specific feedback on all categories. What are they doing well, and what not, how can you improve this.

Klik hieronder op een categorie voor meer informatie over dit product en suggesties voor veranderingen die ú zou kunnen maken in die categorie. U kiest en bepaalt uiteindelijk zelf wát u wilt veranderen. Kies voor een verandering die voor u aantrekkelijk en haalbaar is en goed in te passen in uw dagelijks leven.

Klik hier om meer te lezen over uw zuivel inname

Klik hier om meer te lezen over uw broodbeleg inname

BROODBELEG

U belegt uw brood vooral met beleg waar veel calorieën in zitten en die dus veel energie leveren. Hieronder geven we een overzicht van het beleg dat u meestal kiest. Ook krijgt u informatie over de veranderingen die u zou kunnen maken om GRIP op uw gewicht te krijgen.

20+ en 30+ kazen

U gebruikt meestal normale porties 20+ of 30+ kaas op brood. Wat soort betreft is deze kaas de beste keuze. Aan de andere kant levert ook deze kaas relatief veel energie. Twee keer zoveel, bijvoorbeeld, als een belegging jam of honing.

Als u toch iets wilt veranderen aan uw broodbeleg, kunt u er voor kiezen om kleinere porties kaas te gebruiken of kaas (af en toe) te vervangen door andere soorten broodbeleg, zoals jam, honing, rauwkost of fruit. Een boterham besmeerd met 20+ smeerkaas levert trouwens al minder energie dan een boterham belegd met 'gewone' 20+ kaas.

Tip: De termen jong, jong belegen, belegen, oud etc. slaan op de leeftijd van de kaas. Niet op de hoeveelheid energie of vet.

Chocolade beleg
Regelmatig doet u chocolade beleg op uw brood. Dit beleg bevat veel calorieën en levert dus veel energie. Het bevat bijvoorbeeld evenveel calorieën als een boterham met gewone kaas en twee keer zoveel calorieën als een boterham met jam.

Welke verandering kunt u maken?

- U kunt er voor kiezen om minder chocolade beleg op een boterham te doen. Smeer/strooi bijvoorbeeld dunner, of neem een dubbele boterham met enkel beleg.
- U kunt er ook voor kiezen om minder vaak chocolade beleg te eten. U kunt het vervangen door magere vleeswaren, rauwkost, plakjes fruit of zoet beleg zoals jam, honing of appelstroop.

Pindakaas
U besmeert uw brood vaak met pindakaas. Het is weliswaar zo dat pindakaas goede vetsoorten bevat, maar daar zitten óók veel calorieën (en dus veel energie) in. Een boterham met pindakaas levert nog meer calorieën dan een boterham met 'gewone' kaas en bijna drie keer zoveel als een boterham met appelstroop.

Mogelijkheden voor een kleine verandering

- Als u iets wilt veranderen aan uw broodbeleg dan kunt u er voor kiezen om minder vaak pindakaas te eten.
- U kunt er ook voor kiezen om minder veel pindakaas te eten, door dunner te smeren, of een dubbele boterham met enkel beleg te nemen.

Zoet beleg
Regelmatig zoet beleg eten, is zo gek nog niet. Ondanks het feit dat er suiker in zit, bevatten jam, honing en appelstroop veel minder calorieën dan bijvoorbeeld chocopasta, pindakaas of kaas. Een gezond alternatief voor zulk zoet beleg is fruit of rauwkost op uw brood. Mocht u iets aan het beleg op uw brood willen doen, dan kunt u er eventueel ook voor kiezen de grote porties zoet beleg die u neemt te verkleinen.

	**Veel energie**	**Middenweg**	**Weinig energie**
**Overige broodbeleg**	Chocoladepasta, hazelnootpasta, kokosbrood, pindakaas	Chocoladehagelslag, chocoladevlokken, vruchtenhagel	groentespread, (halva-)jam, honing, (appel-)stroop

Altijd goed: Fruit (plakjes appel, banaan, aardbeien, etc.), groente (alfalfa, komkommer, radijs, tuinkers, tomaat, etc.)

Then, the participant scan choose a goal, set it, and make a plan.

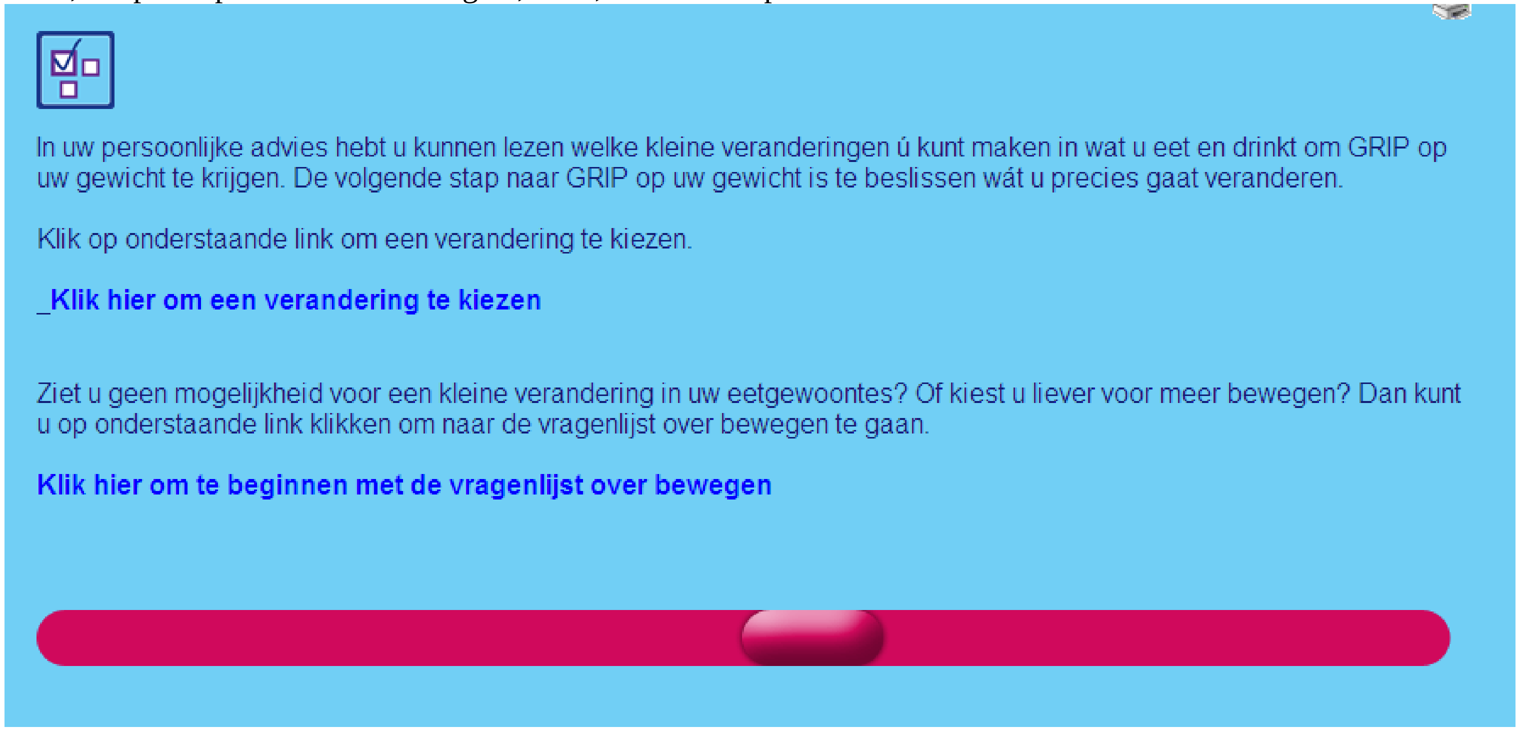

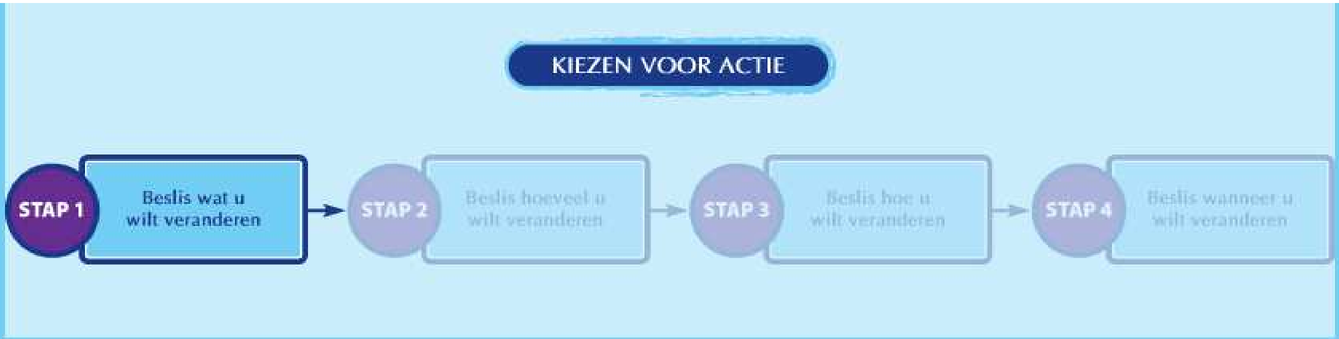

Stap 1: Beslis wat u wilt veranderen

Uit de antwoorden die u gaf op onze vragen blijkt dat u een aantal kleine veranderingen zou kunnen aanbrengen in uw beweeg- en/of voedingspatroon. Wij hebben deze veranderingen voor u op een rijtje gezet, zodat u er één kunt kiezen voor de komende week. Als u deze verandering goed doorvoert en er verder voor zorgt dat u niet meer of anders gaat eten of minder gaat bewegen, dan zal deze verandering zorgen voor meer gewichtscontrole.

Om de kans op succes te vergroten, is het goed om een verandering te kiezen die u prettig vindt en die u dagelijks kunt doorvoeren. Kiest u voor een beweegverandering, dan is het verder belangrijk om deze verandering minstens 20 minuten per dag vol te houden. Dat hoeft niet 20 minuten achter elkaar te zijn.

Klik nu op de verandering die u de komende week wilt doorvoeren.

Let op: als u per ongeluk een verkeerde keuze heeft gemaakt, dan kunt u die ongedaan maken en opnieuw kiezen.

Ik kies ervoor om kleinere porties aardappels-rijst-pasta te eten

Ik kies ervoor om minder suikerrijke dranken te drinken

Ik kies ervoor om minder calorierijk broodbeleg te eten

Ik kies ervoor om minder calorierijke vlees, vis of vleesvervangende producten te eten

Ik kies ervoor om minder tussendoortjes te eten

Uw doel is om minder calorierijk broodbeleg te eten.

Hoeveel wilt u veranderen?

Hoeveel minder boterhammen per dag wilt u beleggen met calorierijk broodbeleg?

- 1 boterham
- 2 boterhammen
- 3 boterhammen
- 4 boterhammen
- 5 boterhammen
- Meer dan 5 boterhammen

Let op: het is belangrijk dat u een haalbare hoeveelheid kiest.

Op welke manier wilt u veranderen?

Hoe wilt u minder calorierijk broodbeleg gaan eten?

- Door dit vervangen door een gezonder alternatief (bijvoorbeeld 20+ kaas)
- Door de boterhammen weg te laten

Wanneer wilt u veranderen?

Op welk *moment* (of momenten) van de dag wilt u de kleine verandering doorvoeren? Let er wel op dat u een moment beschrijft dat zich minimaal 5 keer in de week voordoet. U kunt dan bijvoorbeeld denken aan de volgende momenten:

-Als ik aan het ontbijten ben
-Als ik lunch
-Als ik tussendoor trek krijg
-Als het koffietijd is

Uw doel ziet er momenteel als volgt uit:

Als ik ontbijt en lunch dan ga ik 3 boterhammen met calorierijk broodbeleg vervangen door een gezonder alternatief (bijvoorbeeld 20+ kaas).

Staat u helemaal achter dit doel?

- (•) ja
- ( ) nee

Wat kunt u nog doen om goed voorbereid te zijn op uw kleine verandering?

Een goede voorbereiding verhoogt uw kans op succes. bedenk daarom of u helemaal klaar bent om aan de slag te gaan met uw kleine verandering. U gaat bijvoorbeeld een lijstje maken met de spullen die u nog in huis moet halen (zoals gezonde producten), nadenken over of u bij het maken van uw kleine verandering steun wilt van familie of vrienden, en uzelf alvast voorbereiden op moeilijke situaties.

Wij kunnen u helpen om goed voorbereid aan de slag te gaan aan de hand van een drietal stappen. De stappen die u dan heeft doorlopen kunt u op het einde, in de vorm van een voorbereidingsplan, afdrukken.

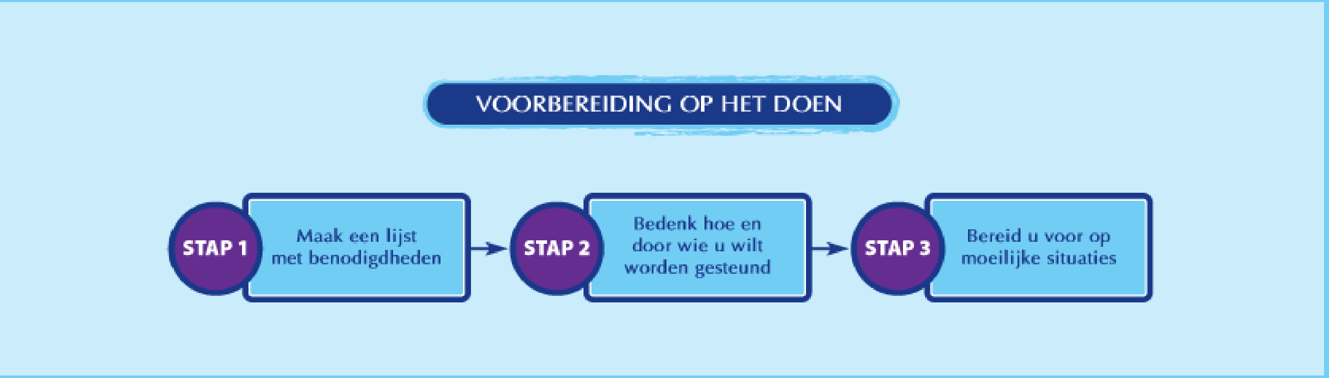

Bij welke stappen wilt u graag hulp van dit programma?

1. Leren hoe u kunt bepalen of een product gezond is zodat u een lijst met benodigdheden kunt maken

○ Ja

◉ Nee

2. Bedenken hoe en door wie u gesteund wilt worden

○ Ja

◉ Nee

3. Uzelf voorbereiden op moeilijke situaties

○ Ja

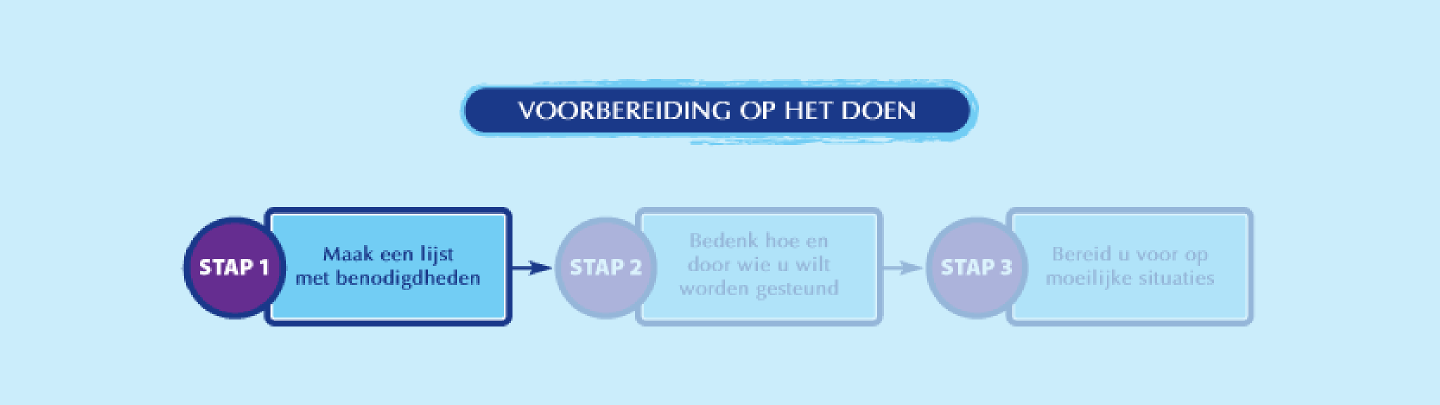

De juiste spullen in huis

U hebt aangeven dat u graag wat hulp wilt bij het maken van een lijst met benodigdheden. Omdat u een eetverandering gaat maken, is het vooral belangrijk om alvast na te denken over de calorie-armere alternatieven die bij uw kleine verandering passen. Daarvoor moet u natuurlijk wel weten hoe u kunt bepalen of een product calorie arm is.

De meest betrouwbare manier om dit te doen is door het voedingsetiket op het product te lezen. Wilt u weten hoe een etiket eruit ziet en wat voor informatie een etiket bevat, klik dan op het kopje 'voedingsetiketten'.

Wilt u direct weten wat voor u gezonde alternatieven zijn, klik dan op het kopje 'gezonde alternatieven'.

Klikt u op 'portiegrootte'', dan kunt u meer lezen over wat geschikte portiegroottes zijn.

Hoe kom erachter hoeveel calorieën een product bevat?

Op elk voedingsproduct zit tegenwoordig een etiket. Dat ziet er zo uit:

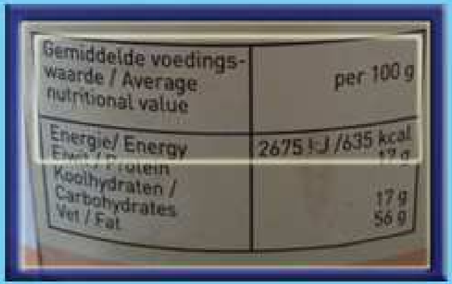

Op dit etiket staat onder andere hoeveel calorieën het product bevat per 100 gram of per portie.
Hieronder ziet u de etiketten van pindakaas en jam.

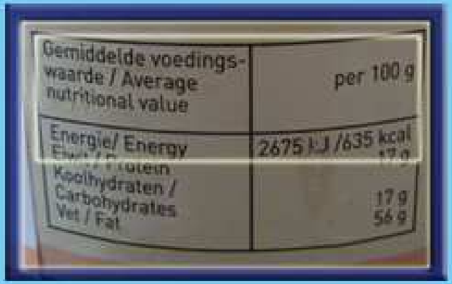

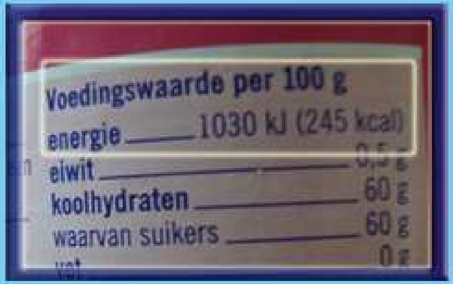

Kunt u zien wat het verschil is in aantal calorieën tussen 100 gram pindakaas en 100 gram jam?

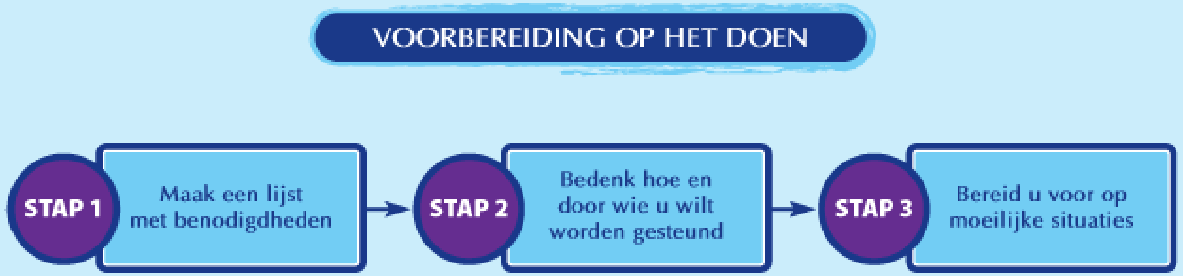

Wat heeft u nog nodig?

Om uw verandering dagelijks door te kunnen voeren is het belangrijk om de juiste en gezonde spullen in huis te halen. Maak daarom nu alvast een lijstje van de benodigdheden voor uw verandering.

TIP: dit hoeven niet alleen voedingsproducten te zijn maar kunnen bijvoorbeeld ook kleinere schaaltjes/borden/glazen of andere hulpmiddelen zijn.

- een pot appelstroop
- magere smeerkaas (bijv. Slankie)

Let op: Als u alle benodigdheden al in huis heeft dan kunt u "geen" invullen

The above questions lead to a combined plan:

GO!

Uw plannen voor de komende week:

Uw doel:

Als ik avondeet dan ga ik dagelijks 50 gram rijst, pasta of aardappelen minder eten door minder aardappels op te scheppen bij warme maaltijd

---

Uw doel:

Als ik ontbijt en lunch dan ga ik 3 boterhammen met calorierijk broodbeleg vervangen door een gezonder alternatief (bijvoorbeeld 20+ kaas).

Uw benodigheden en voorbereidingen:

- een pot appelstroop - magere smeerkaas (bijv. Slankie)

Doe alsof
Lees dit plan een paar keer goed door. Als u denkt dat u het kent, sluit dan uw ogen, en stelt u zich eens die moeilijke situatie voor en hoe u bovenstaand actieplan dan zult uitvoeren. Door dit in gedachten de situatie al te beleven, kunt u in de moeilijke situatie nog sneller reageren.
Als u duidelijk voor ogen hebt wat u in een moeilijke situatie gaat doen, wordt het gemakkelijker om daarmee om te gaan.

Herinnering
Wilt u een extra geheugensteuntje? U kunt uw plan ook uitprinten (klik hiervoor op de afbeelding van de printer) en bijvoorbeeld op de koelkast plakken of in uw tas stoppen, zodat u het altijd terug kunt zien.

Verwacht u nog meer moeilijke situaties?
Op deze manier kunt u zelf ook plannen maken voor andere moeilijke situaties. Bedenk wat voor u een moeilijke situatie is, en hoe u hier mogelijk mee om kan gaan.

Tip: Door dit overzicht uit te printen, heeft u duidelijk op een rijtje wat u wilt bereiken en hoe u dit wilt aanpakken.

Module 2:

Welkom terug bij GRIPP!

De vorige keer heeft u een kleine verandering gekozen. Vandaag kunt u in het GRIP programma KIJKEN hoe dit gegaan is. U kunt beschrijven wat voor u moeilijke momenten waren, en hoe u deze een volgende keer kunt oplossen. Als u wilt kunt u een nieuwe, of extra verandering kiezen. U zult ongeveer 15 minuten bezig zijn met dit bezoek.

Wat is de datum waarop u zich voor een tweede keer gewogen hebt. Geef dit a.u.b. alleen in getallen weer. Bijvoorbeeld 19-10 voor 19 oktober.

30-4

Wat was toen uw gewicht?

84

Tijdens uw vorige bezoek heeft u voor het volgende doel gekozen:

Als ik ontbijt en lunch dan ga ik 3 boterhammen met calorierijk broodbeleg vervangen door een gezonder alternatief (bijvoorbeeld 20+ kaas).

Houdt u dit doel in gedachten bij het beantwoorden van de vragen op de volgende pagina's.

De afgelopen week heeft u geprobeerd de door u gekozen verandering te maken. Geef hieronder aan op welke dag u daar wel, niet of gedeeltelijk in geslaagd bent.

	Ja, helemaal	Ja, gedeeltelijk	Nee, niet.
Heeft u gisteren (1 dag geleden) de door u gekozen verandering kunnen maken?	●	○	○
Heeft u eergisteren (2 dagen geleden) de door u gekozen verandering kunnen maken?	○	●	○
Heeft u 3 dagen geleden de door u gekozen verandering kunnen maken?	○	○	●
Heeft u 4 dagen geleden de door u gekozen verandering kunnen maken?	○	●	○
Heeft u 5 dagen geleden de door u gekozen verandering kunnen maken?	●	○	○
Heeft u 6 dagen geleden de door u gekozen verandering kunnen maken?	○	●	○
Heeft u 7 dagen geleden de door u gekozen verandering kunnen maken?	○	○	●

Beste Linda,

Afgelopen week heeft u geprobeerd om het volgende doel dagelijks uit te voeren.
Als ik ontbijt en lunch dan ga ik 3 boterhammen met calorierijk broodbeleg vervangen door een gezonder alternatief (bijvoorbeeld 20+ kaas).

Hieronder kunt u zien hoe dat gegaan is, vanaf 7 dagen geleden tot nu.

✓ betekent: verandering *gemaakt*.

● betekent: verandering *gedeeltelijk* gemaakt.

✗ betekent: verandering *niet* gemaakt.

Wanneer	Verandering gemaakt	Gedeeltelijk gemaakt	Niet gemaakt
Gisteren	✓		
Eergisteren		●	
3 dagen geleden			✗
4 dagen geleden		●	
5 dagen geleden	✓		
6 dagen geleden		●	
7 dagen geleden			✗

Uit het schema blijkt dat het u in de afgelopen zeven dagen een paar dagen is gelukt om de door u gekozen eet-verandering te maken. Daar mag u best trots op zijn. U hebt voor een deel gedaan wat u zich had voorgenomen en daarmee een eerste stap in de goede richting gezet. Let u er wel op dat u - op de dagen dat u minder calorierijk eet dan voorheen - niet minder gaat bewegen of meer gaat snoepen dan u normaal deed?

In de afgelopen zeven dagen is het u niet altijd gelukt om uw kleine verandering te maken. Daar hoeft u zich absoluut niet door uit het veld te laten slaan. Het veranderen van een vast leefpatroon gaat 'met vallen en opstaan'. Hieronder gaan we in op wat u kunt doen om ervoor te zorgen dat u ook op de andere dagen de gekozen verandering kunt maken.

In de afgelopen zeven dagen is het u een paar keer niet gelukt om de door u gekozen eet-verandering (helemaal) te maken.
Gaat u in gedachten eens terug naar de laatste keer dat het niet lukte.

Wat vond u er van toen het niet lukte? En hoe voelde u zich daarbij?

ik baalde, voel me rot.

Hielpen deze gedachten u om de volgende dag de eetverandering wél (helemaal) te maken?

Ja Nee

○ ◉

Het belang van onze gedachten

Hoe u denkt en wat u voelt is van invloed op het maken van de verandering. Positief denken is hierbij heel belangrijk. In de volgende oefening gaat u kijken hoe u dat kunt doen.

Niet alle gedachten zijn behulpzaam

In bepaalde situaties kan het moeilijk zijn een verandering te maken. Wat u denkt en voelt bij een situatie kan echter bepalend zijn voor hoe u met een situatie omgaat.
We kunnen hier 2 type gedachten onderscheiden:
niet helpende gedachten
helpende gedachten.

Niet helpende gedachten helpen u niet om de verandering de volgende dag wél te maken, en zorgen ervoor dat u uw vertrouwen verliest.

Voorbeelden van niet-helpende gedachten en gevoelens
'Het lukt me *nooit*'<br>*'Iedereen* vindt me lui'<br>'Het gaat *altijd* mis'

Helpende gedachten

Helpende gedachten zijn **positief** en **realistisch.** Zij helpen u om beter met een moeilijke situatie, die mogelijk niet te voorkomen is, te gaan.

Voorbeelden van helpende gedachten en gevoelens
'Ik *kan* het wel, maar door die moeilijke situatie lukte het even niet.'<br>'Jammer, het is vandaag niet gelukt. *Morgen* probeer ik het nog een keer!'<br>'Van fouten kun je *leren*.'

'Van fouten kun je *leren*.'

U kiest wat u denkt!

Zoals gezegd: Sommige moeilijke situaties zijn niet te vermijden. U hebt echter wel controle over wat u denkt. Kies daarom gedachtes, die u kunnen helpen in zo'n situatie.

Ga nu nog eens terug naar de laatste situatie waarin het u niet lukte een kleine verandering te maken: Wat zou voor u een helpende gedachte zijn?

U kunt zelf een gedachte bedenken of er eentje kiezen uit de voorbeelden hierboven.

Als het me een keer niet lukt om de verandering te maken, dan denk ik:

U zult zien dat er na deze positieve gedachten ook positieve gevoelens optreden, waardoor het een stuk makkelijk is om de verandering te blijven maken. Want wat u denkt, heeft grote invloed op wat doet. Want daar gaat het uiteindelijk om, dat u de kleine verandering ook echt maakt.

U bepaalt wat u doet

En wat gaat u dóén als de verandering een keer niet lukt? Gaat u het later op de dag of de dag erna gewoon weer proberen?
Beschrijf hieronder wat u gaat doen.
Als het me een keer niet lukt om de verandering te maken, dan:

U hebt nu uw eigen plan gemaakt, mét positieve gedachten voor als het u een keertje niet lukt om de verandering te maken. Dit plan ziet er als volgt uit:

Gebeurtenis	Gedachte/gevoel	Gevolg
Het is me vandaag niet gelukt om de verandering te maken.	Niet-helpende gedachte/gevoel: *ik baalde, voel me rot.*	De dag erna was het weer moeilijk of zelfs onmogelijk om de verandering te maken
Als het me nog een keer niet lukt om de verandering te maken,	dan denk ik: *dat is balen, maar het kan iedereen overkomen.*	en dan: *zal ik nadenken wat er mis ging, en hoe ik het volgende keer beter kan doen.*

Lees dit plan een paar keer goed door. Als u dan in deze situatie komt, wordt het gemakkelijker om te reageren zoals ú dat wilt.

Plan van aanpak voor moeilijke situatie

Het veranderen van een vast leefpatroon gaat met vallen en opstaan. Het is een leerproces. Dát het mis gaat, ligt vaak aan de situatie. In sommige situaties is het nu eenmaal moeilijk om je 'goede' nieuwe gedrag vol te houden. Van de keren dat het mis ging, kunt u leren. Door goed te kijken naar wat er precies mis ging en te bedenken wat u anders zou kunnen, voorkomt u dat het in de volgende keer dat u in deze moeilijke situatie komt weer misgaat. We zetten de stappen die u gaat nemen even voor u op een rijtje:

1. Bedenk wanneer het u niet lukte om uw verandering te maken.
2. Bedenk wat u de volgende keer in deze situatie zou kunnen doen om ervoor te zorgen dat u uw nieuwe gedrag wel volhoudt.

Op deze manier ontwikkelt u een persoonlijk actieplan.

Ga in gedachten terug naar een van de keren in de afgelopen week dat het u niet lukte om de kleine verandering te maken. Dat kan de laatste keer zijn, of de keer die u zich het beste herinnert.
Welke van onderstaande beschrijving past daar het best bij?

- ○ Het gezonde product was niet voorhanden.
- ○ Ik of iemand anders had op dat moment iets te vieren (bijvoorbeeld een traktatie) of ik wilde mezelf belonen.
- ○ Ik voelde me rot, bijvoorbeeld omdat ik verdrietig, gestresst, angstig, verveeld of eenzaam was.
- ◉ Ik had een heel sterk verlangen om (een bepaald) iets te eten, bijvoorbeeld na een reclame of het ruiken van iets.
- ○ Ik was samen met anderen, bijvoorbeeld op een feestje, tijdens het eten of een uitje.
- ○ Ik kon me er niet tot zetten
- ○ Deze beschrijvingen zijn niet van toepassing

Uw persoonlijke plan van aanpak

U heeft zich vorige week voorgenomen om dit doel te bereiken:
Als ik ontbijt en lunch dan ga ik 3 boterhammen met calorierijk broodbeleg vervangen door een gezonder alternatief (bijvoorbeeld 20+ kaas).
Als ik avondeet dan ga ik dagelijks 50 gram rijst, pasta of aardappelen minder eten door minder aardappels op te scheppen bij warme maaltijd.
Uw persoonlijke plan ziet er als volgt uit:

De volgende keer ***als ik sterk verlang naar een bepaald product,*** *dan ga ik:* ***afleiding zoeken als ik heel veel trek heb in een boterham brie. Ik zorg ook dat er een lekker alternatief is, bijvoorbeeld smeerkaas met wat tuinkers.***

Doe alsof
Lees dit plan een paar keer goed door. Als u denkt dat u het kent, sluit dan uw ogen, en stelt u zich eens die moeilijke situatie voor en hoe u bovenstaand actieplan dan zult uitvoeren. Door dit in gedachten de situatie al te beleven, kunt u in de moeilijke situatie nog sneller reageren.
Als u duidelijk voor ogen hebt wat u in een moeilijke situatie gaat doen, wordt het gemakkelijker om daarmee om te gaan.

Herinnering
Wilt u een extra geheugensteuntje? U kunt uw plan ook uitprinten (klik hiervoor op de afbeelding van de printer) en bijvoorbeeld op de koelkast plakken of in uw tas stoppen, zodat u het altijd terug kunt zien.

Verwacht u nog meer moeilijke situaties?
Op deze manier kunt u zelf ook plannen maken voor andere moeilijke situaties. Bedenk wat voor u een moeilijke situatie is, en hoe u hier mogelijk mee om kan gaan.

Module 3 is very similar to module 3.

Module 4

Welkom terug bij GRIPP, voor uw vierde en laatste bezoek!

De afgelopen weken hebt u gewerkt aan het onder controle krijgen van uw gewicht door:

1. te KIJKEN naar uw eet- en beweegpatroon;
2. te KIEZEN voor een kleine verandering en u goed voor te bereiden op het maken van die verandering
3. te DOEN wat u zich hebt voorgenomen.

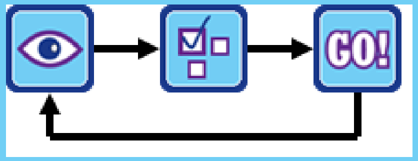

Dit bezoek staat in het teken van de afsluiting en zal u helpen om straks zélf uw gewicht onder controle te houden. Eerst kunt u kijken hoe het er voor staat met uw gewicht, en leert u hoe u dit in de gaten kunt houden. De stappen van KIJKEN, KIEZEN en Doen worden nog eens op een rijtje gezet, zodat u deze stappen in de toekomst zelf kunt toepassen. Tenslotte kunt u een persoonlijk certificaat uitprinten met uw doelen en plannen.

Wat is de datum waarop u zich voor een vierde keer gewogen hebt? Geef dit a.u.b. alleen in getallen weer. Bijvoorbeeld 19-10 voor 19 oktober.

2-3-09

Wat was toen uw gewicht?

86

Hoe behoud ik mijn gewicht?

Door regelmatig te KIJKEN naar of uw kleine verandering gelukt is, wordt duidelijk of het goed gaat
Om uw gewicht te behouden is het belangrijk om ook regelmatig naar uw gewicht te KIJKEN. U komt er dan namelijk achter of de door u gekozen verandering zorgt voor een stabiel gewicht.

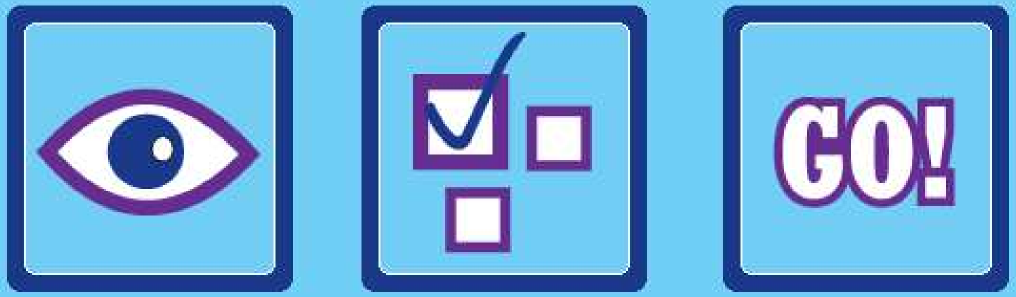

De KIJKEN, KIEZEN en DOEN stappen kunnen u ook helpen om uw gewicht te behouden, en in actie te komen als dat nodig is. Het is dus belangrijk dat u zich deze stappen eigen maakt, om zo ook in de toekomst te voorkomen dat u aankomt.

Op de volgende pagina's hebben wij alle stappen die belangrijk zijn voor gewichtsbehoud voor u op een rijtje gezet.

KIJKEN naar uw gewicht

Om erachter te komen of u uw gewicht daadwerkelijk onder controle hebt, is het belangrijk om wekelijks naar uw gewicht te KIJKEN.

De afgelopen weken hebt u uw gewicht ingevoerd in het programma GRIPP. De blauwe lijn in onderstaande grafiek geeft het verloop van uw gewicht weer.

Een rechte lijn duidt op een stabiel gewicht. Maar in werkelijkheid schommelt ons gewicht altijd een beetje: het ligt iets hoger als we net gegeten hebben, iets lager als we net gesport of geslapen hebben en ga zo maar door. Deze uiterste waarden van uw gewicht noemen we de boven- en ondergrens. In de grafiek hieronder kunt u uw persoonlijke onder- en bovengrens beKIJKEN.

Uw gewichtsverloop de afgelopen 3 weken

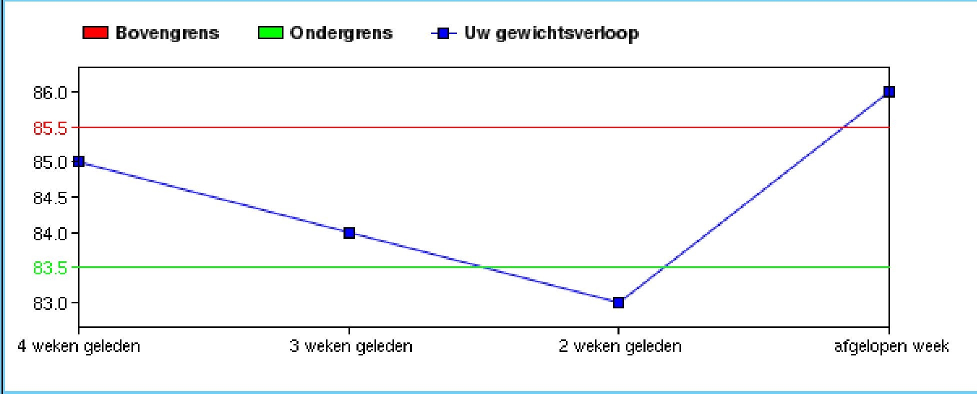

Uw gemiddelde gewicht

Aan de hand van de cijfers die u de afgelopen vier weken hebt ingevoerd, hebben we uw gemiddelde gewicht berekend. Dat is: **84.5 kg**.

Als uw gewicht stabiel is, ligt het altijd rond dit gemiddelde en dus ook binnen de twee grenzen. Uw gemiddelde bovengrens is **85.5 kg** en uw gemiddelde ondergrens is **83.5 kg**. In deze grafiek worden deze grenzen aangegeven met een rode en een groene lijn.

Kijkt u zelf eens goed naar de blauwe lijn, en hoe deze ligt ten opzichte van de groene en rode lijn. Wat valt u op?

Gewichtscontrole: uw gewicht onder de rode lijn

Zolang uw gewicht lager is dan 85.5 kg, heeft u uw gewicht onder controle. U komt dan immers niet aan.
Als uw gewicht lager is dan 83.5 kg (de groene lijn) dan bent u zelfs een beetje afgevallen.

Als u merkt dat uw gewicht langere tijd onder de rode lijn blijft, kunt er ervoor kiezen om om u minder vaak te wegen. Bijvoorbeeld, 1 keer in de twee weken of 1 keer per maand.

Om ook in de toekomst uw gewicht onder controle te houden is het belangrijk om dagelijks de door u gekozen kleine verandering te blijven maken én uw gewicht in de gaten te blijven houden.

Boven de rode lijn: toename van uw gewicht

Komt u er bij het wegen achter dat u een keer meer weegt dan 85.5 kg weegt? Dit betekent dat u wat bent aangekomen. Dit kan verschillende oorzaken hebben.

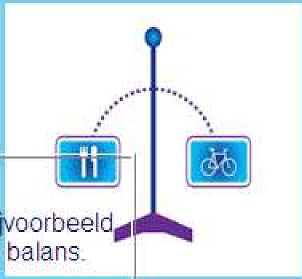

Mogelijke oorzaken van gewichtstoename

Als u bent aangekomen, is uw energiebalans niet in balans. Dit kan 2 oorzaken hebben:

1. *Compensatie*:
U hebt het effect van de door u gekozen verandering (wellicht ongemerkt) teniet gedaan door bijvoorbeeld minder te gaan bewegen of meer te gaan eten of drinken. Uw energiebalans is dus niet meer in balans.

Herkent u dit? Blijf dan de gekozen verandering maken, maar doe daarnaast weer wat u altijd deed. Beweeg dus weer iets meer of eet/drink weer wat minder.

2. *De door u gekozen verandering heeft geen of te weinig resultaat opgeleverd*:
Als u - naast de kleine verandering - niets hebt veranderd aan uw eet- of beweegpatroon, dan is uw energiebalans nog niet helemaal in evenwicht. U krijgt dus nog steeds meer calorieën binnen (door eten en te drinken) dan u verbruikt (door te bewegen en sporten).

Herkent u dit? KIES dan een extra verandering
Door nog een kleine verandering te maken, kunt u uw energiebalans meer in evenwicht brengen. U hebt al eerder met succes een kleine verandering gemaakt. Het gaat u dus vast lukken om nog een kleine verandering te maken.

In het kort: KIJKEN naar uw gewicht

We hebben de verschillende stappen van KIJKEN naar uw gewicht nog eens voor u op een rijtje gezet.

1. **Weeg uzelf** één keer per week. Vaker wegen levert een onbetrouwbaar beeld op en kan ontmoedigend zijn. Blijft uw gewicht een langere tijd stabiel dan kunt u ook overgaan op 1 keer in de 2 weken wegen of 1 keer per maand.

2. KIJK of uw gewicht tussen uw 'grenzen' voor een stabiel gewicht ligt, dus **tussen de 83.5 kg en 85.5 kg**.

3. KIJK of u er een **extra verandering** bij moet KIEZEN

- Ligt uw gewicht tussen de grenzen, dan bent u op gewicht gebleven. Ga dan op de huidige manier door. U kunt ook nog een extra verandering kiezen. Misschien valt u dan een beetje af. Als u verder gaat, leest u hoe u dat kunt doen.
- Ligt **uw gewicht boven uw bovengrens**, dan bent u een beetje aangekomen. KIJK dan waar dat aan ligt. Levert de door u gekozen verandering geen of te weinig resultaat op? **Kies in dat geval een nieuwe of een extra verandering**. Als u verder gaat, leest u hoe u dat kunt doen.

Kijken, kiezen en doen dus!

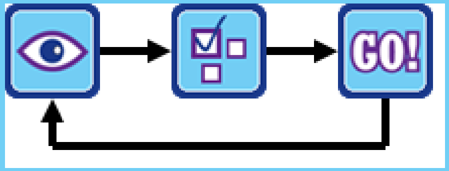

Het KIEZEN van een nieuwe verandering

Als u een beetje bent aangekomen, is het nodig om een extra verandering te KIEZEN. U kunt ook een extra verandering kiezen als u op gewicht bent gebleven. Misschien valt u dan een beetje af.

Met zorg KIEZEN

KIES deze nieuwe of extra verandering met zorg:

1. Bedenk (of maak een lijstje van dingen) welke verandering in uw eet- of beweeggedrag echt zal bijdragen aan het in evenwicht brengen van uw energiebalans.
2. KIES hieruit een kleine verandering die u zou kúnnen en willen maken: Een verandering die u niet teveel moeite kost, die u denkt te kunnen volhouden en die u plezierig vindt.

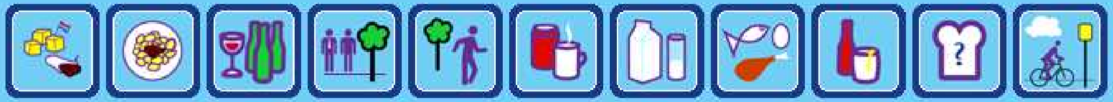

3. Maak deze kleine verandering concreet: Bedenk hoeveel minder u wilt eten of drinken, of op welke manier u meer gaat bewegen. Kiest u voor een gezonder alternatief of een kleinere portie? Gaat u meer wandelen, fietsen of iets anders doen? En doet u dat alleen of met iemand samen?

Kortom:

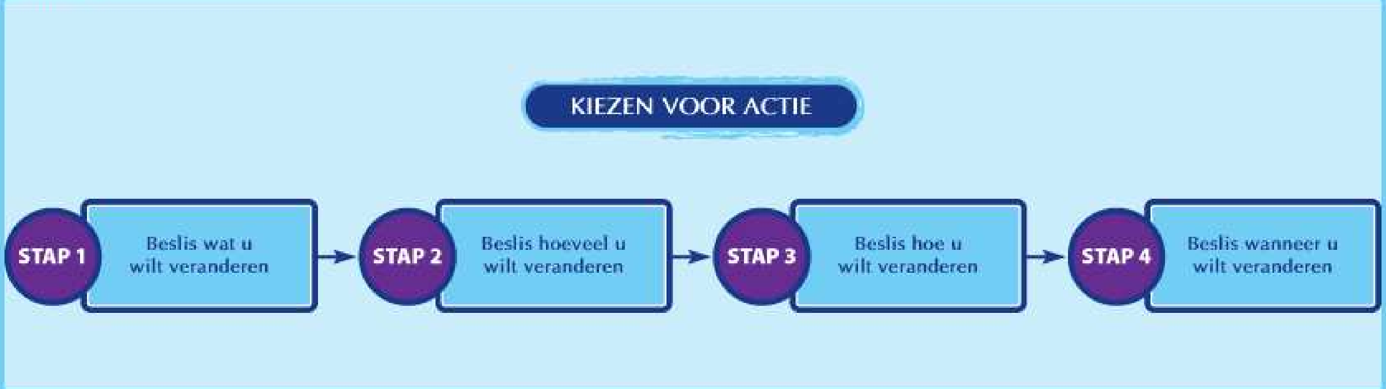

Een goede voorbereiding

KIES met zorg welke voorbereiding u nodig heeft:

1. Tref alle noodzakelijke voorbereidingen. Zorg ervoor dat u de dingen in huis hebt die u nodig hebt (gezonde producten, sportschoenen) of dat u bijvoorbeeld een afspraak hebt gemaakt met uw beweegmaatje.
2. Kunnen de mensen in uw omgeving u helpen om de kleine verandering te maken? Vraag of ze u willen helpen.
3. Bedenk in welke situaties het moeilijk zou kunnen zijn om de door u gekozen verandering te maken. Waar zou dat aan kunnen liggen? Wat zou u kunnen doen om ervoor te zorgen dat u de kleine verandering wel maakt? Als u van tevoren een plan maakt, wordt het gemakkelijker om de verandering ook echt elke dag te maken.

Kortom:

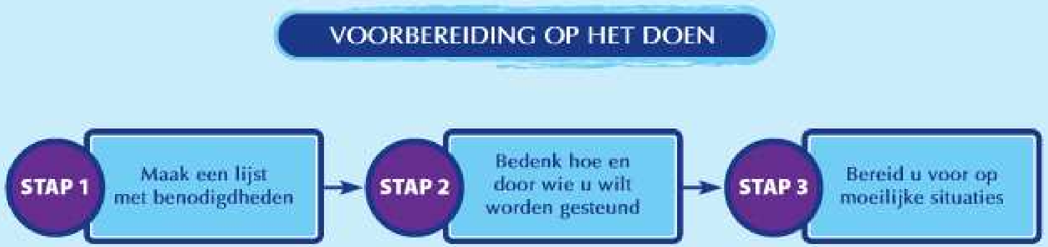

In het kort: KIEZEN van een verandering
1. KIJK naar wat en hoeveel u eet/drinkt en naar hoe en hoeveel u beweegt.
2. Bedenk wat u kunt en wilt veranderen en KIES een verandering
3. Bereid u voor: Wat moet u nog in huis halen of regelen? En wat gaat u doen in moeilijke situaties?

U bent nu klaar om te DOEN!

Doen!

Rekening houden met eventuele tegenslagen

Als u straks bezig bent met uw nieuwe leefwijze kunt u te maken krijgen met tegenslagen. Het is verstandig om daar rekening mee te houden, zodat u zich er niet door uit het veld laat slaan. Er zullen zich bijvoorbeeld zo nu en dan situaties voordoen waarin het u niet lukt de kleine verandering te maken. Of u maakt de kleine verandering wel, maar komt toch een beetje aan.

Denk bij tegenslagen aan het volgende:

1. U bent bezig met het doorbreken van een gewoonte. Die gewoonte is niet in een paar weken ontstaan. Het doorbreken van zo'n gewoonte gaat daarom ook (vaak) niet 'zomaar'.
2. Houd voor ogen waarom u de kleine verandering wilde maken. Doe dat ook als het maken van de verandering u wél gemakkelijk afgaat.

Aan het begin van het programma zei u dat het maken van de kleine verandering voor u de volgende gevolgen heeft:
Dan word ik niet zwaarder
Geen

3. Gaat het een keer mis, denk dan aan hoe u zich voelde toen het u wel lukte de verandering te maken. Het zal u motiveren de draad weer op te pakken.
4. Dat het een keertje niet lukt om de verandering te maken, is heel normaal. Laat u er dus niet door ontmoedigen. Het is wel belangrijk om even stil te staan bij waarom het nu (even) niet lukte. Bedenk verder dat het u in heel veel situaties wél is gelukt de door u gekozen verandering te maken. U kunt het dus echt wel!

Leren van tegenslagen

Zit het een keer tegen, bedenk dan of u:
- deze situatie de volgende keer kunt vermijden;
- de volgende keer anders met deze situatie kunt omgaan;
- de moeilijke situatie de volgende keer kunt zien aankomen, zodat u tijdig kunt bedenken wat u kunt doen opdat u de kleine verandering toch kunt maken.

Beloon uzelf
Het is niet gemakkelijk om een gewoonte te doorbreken. U kunt het wat makkelijker maken door uzelf te belonen als het u bijvoorbeeld een week lang lukt om de kleine verandering te maken. U kunt een mooie cd kopen voor uzelf, of een grote bos bloemen. Of neem eens wat extra tijd voor uzelf, bijvoorbeeld om dat ene boek uit te lezen. U zult zien dat het u motiveert om door te gaan.

Hoe gaat u zichzelf belonen? Beschrijf wanneer u zichzelf gaat belonen (bijvoorbeeld: als het 2 weken gelukt is om de verandering te maken) en hoe (bijvoorbeeld: door mijn favoriete film te kijken).

Ik beloon mezelf:

als ik 2 maanden lang niet zwaarder ben geworden, door een leuk boek te kopen

In het kort: DOEN
1. Probeer het gewoon!
2. Gaat het een keer mis, bedenk dan waar dat aan lag en hoe het een volgende keer beter kan.
3. Beloon uzelf!
4. Lukt het toch allemaal niet? Laat u dan niet uit het veld slaan en probeer het de volgende dag gewoon weer.

GRIPP CERTIFICAAT
Linda Heeft met succes GRIPP afgerond. Ten bewijze van de succesvolle afronding wordt dit certificaat uitgereikt. Uw doelstellingen nog eenmaal op een rijtje. We hopen dat u hier ook in de toekomst verder mee aan de slag wilt gaan.

Uw afspraken
Ik ga proberen mijn gewicht te behouden door iedere week naar mijn gewicht te KIJKEN. Het is dan belangrijk dat mijn gewicht onder de 85.5 kg blijft. Als ik merk dat mijn gewicht boven de 85.5 kg ligt dan ga ik een extra verandering KIEZEN die ik kán en wíl maken. Vervolgens ga ik wat ik me heb voorgenomen ook echt DOEN!

Binnen het programma heb ik het volgende doel(en) voor mezelf gesteld:

Als ik avondeet dan ga ik dagelijks 50 gram rijst, pasta of aardappelen minder eten door minder aardappels op te scheppen bij warme maaltijd.

Als ik ontbijt en lunch dan ga ik 3 boterhammen met calorierijk broodbeleg vervangen door een gezonder alternatief (bijvoorbeeld 20+ kaas).

Ik ga regelmatig KIJKEN of het me is gelukt om dit doel te behalen. Als het me een keer niet lukt, bedenk ik wat er mis ging, en KIES ik een plan om het de volgende keer beter te DOEN.

Ik beloon mezelf als het me lukt om dit doel te behalen, door *als ik 2 maanden lang niet zwaarder ben geworden, door een leuk boek te kopen.*

Ik laat me niet uit het veld slaan als het een keertje niet lukt. Ik blijf positief denken en blijf erop vertrouwen dat ik dit kan. Gaat het een keer mis, dan probeer ik het een dag later gewoon weer. Als dat nodig is, vraag ik mensen in mijn omgeving om me te steunen en/of te helpen.

Als ik twijfel aan of ik hiermee door wil gaan, denk ik aan de door mij genoemde voordelen van gewichtscontrole, namelijk:
1. Dan word ik niet zwaarder
en
2. Geen

Met dit stappenplan blijf ik op gewicht.

Hierbij beloof ik dat ik ga proberen om de in het certificaat beschreven afspraken en plannen na te komen.

Op: 25-4-2012 **Deelnemer:**
Te: Rotterdam Linda M.
