# Supplementary material for: Intervention Use and Action Planning in a Web-Based Computer-Tailored Weight Management Program for Overweight Adults: Randomized Controlled Trial
Source: JMIR Res Protoc. 2014 Jul 23;3(3):e31. doi: 10.2196/resprot.2599 (PMC4129126; doi:10.2196/resprot.2599)
Supplement: Supplementary file 2 [file resprot_v3i2e31_app2.pdf]

Multimedia Appendix 2: Measurement of correlates and outcomes.

**Measure**	**Scale**
**Weight-related behaviour**	
Fat intake	
Fat intake was assessed using a food frequency questionnaire that assessed the frequency and quantity of a variety of high energy foods eaten in the past week. It was based on a validated questionnaire , and it allows the researcher to calculate fat intake in 'fat points'. The questionnaire consisted of 74 questions and was organized according to meal pattern. Participants recorded their frequency of consumption and portion size for a selection of food items eaten during meals or between meals. There were 23 products that fell into the following categories:	
dairy products (5 products)	None, 1, 2, 3, 4, 5, 6, 7+
butter (1)	None, 1, 2, 3, 4, 5, 6, 7+
gravy (1)	None, 1, 2, 3, 4, 5, 6, 7+
sandwich fillings (3)	None, 1, 2, 3, 4, 5, 6, 7+
meat and cheese for main dinner (2)	None, 1, 2, 3, 4, 5, 6, 7+
sweet, salty, hot and cold snacks (11 in total)	None, 1, 2, 3, 4, 5, 6, 7+
Based on daily intake, a 'fat score' was calculated. Higher scores indicate more frequent and/or larger amounts of fat intake.	0-83 points
Physical activity	
Physical activity was assessed using a questionnaire based on the 'Short QUestionnaire to ASsess Health enhancing physical activity' (SQUASH), developed to assess habitual physical activity . In this 16-item questionnaire, participants were asked to indicate how many days of the week they participated in specific activities and how much time they engaged in the activity per occasion.	
Do you walk to your work?	Y/N, how many days/week, how long.
Do you walk to your work?	Y/N, how many days/week, how long.
Do you walk during leisure time?	Y/N, how many days/week, how long.
Do you cycle during leisure time?	Y/N, how many days/week, how long.
What is your sport?	
How often do you perform this sport?	How many days/week, how long.
For each category, the mean number of minutes per day was calculated by multiplying the frequency with the duration and dividing this number by 7. Next, the total number of minutes engaged in physical activity per day was calculated as the sum of all activities (active transportation, leisure time activities and sports).	Mean number of minutes of (total) physical activity per day
**Socio-cognitive variables**	
Intention to prevent weight gain	
Do you intend to prevent weight gain in the next six months?	Definitely not (1) to 'definitely yes' (5)
Perceived behavioral control	
Would you be able to prevent weight gain in the next six months, if you really wanted to?	'definitely not' (1) to 'definitely yes' (5)
Weight locus of control	
Weight Locus of Control (WLOC) scale , 4 item scale. The scale reliability (Cronbach's α) of the four items was 0.61, which is low, but acceptable and comparable to the original scale . Thus, a composite measure (mean value) was created. Cronbach's α of all items was 0.87 and all items were combined to one mean value.	
Whether I gain, lose, or maintain my weight is entirely up to me. (internal)	'totally disagree' (1) to 'totally agree' (5)
Being the right weight is largely a matter of good fortune. (external)	'totally disagree' (1) to 'totally agree' (5)
No matter what I intend to do, if I gain or lose weight or stay the same in the near future, it is just going to happen. (external)	'totally disagree' (1) to 'totally agree' (5)
If I eat properly, and get enough exercise and rest, I can control my weight in the way I desire. (internal).	'totally disagree' (1) to 'totally agree' (5)
Restrained eating	

Restrained sub-scale of the Dutch Eating behavior Questionnaire , 10 item scale.	
E.g. 'After you have put on weight, do you eat less than you usually do?'	'never' (1), 'seldom' (2), 'sometimes' (3), 'often' (4), and 'very often' (5).
Monitoring of weight	
How often did you weigh yourself during the past month?' A dichotomous variable was made for *monitoring of weight*.	Weighting weekly (1) and not weighting weekly (e.g. daily or never) (0).
Planning for PA	
Four items. Cronbach's α was 0.92. Therefore, a composite measures (mean scores) was calculated.	
'Do you have a clear plan for: 1) when, 2) where, 3) how, and 4) with whom you will exercise 20 minutes extra every day?'	'I don't have a plan' (1) to 'I have a very clear plan' (4).
Planning for DI	
Three items. Cronbach's α was 0.94. Therefore, a composite measures (mean scores) was calculated.	
1) 'Do you have a clear plan for what (i.e., which product) to change, 2) how much you will change and 3) when you will make the change?'	'I don't have a plan' (1) to 'I have a very clear plan' (4).
Pro-active coping skills	
21-item 'Proactive Competence Scale' (PCS) which is based on the five phases of coping .. All items were combined in one mean score	
E.g. 'I am able to find solutions'.	1 (not at all able) to 4 (very able)
**Use of the intervention and its components**	
Exposure to the intervention	
An objective measure was obtained by retrieving the log-in data from the intervention server registrations, which registered how often each participant logged on to the program and which intervention modules they visited. Maximum number of log-ins: 0-3 for GI, 0-4 for TI.	Dichotomous *'never-ever' score*: 0 indicates 'never visited' and 1 indicates 'visited at least once' (sum score ≥ 1).
Revisiting the intervention	
For those who visited at least one module (sum score ≥ 1), a dichotomous score was made for *'revisiting'*.	Visited first module only: 0, also visited later modules: 1.
Choose a change in dietary intake and/or physical activity	
Two dichotomous variables were made, indicating whether or not people chose to make a change in dietary intake and/or physical activity.	0: did not chose for DI/PA, 1did choose for DI/PA
Use of the action planning component	
A dichotomous variable was created for *use of the action planning component*¸ based on information from the server registrations.	0: no plan, 1: a plan
*Quality of the goals **	
The quality of the goal was determined, by scoring the text that was written in the text boxes in the program. For this text, one point was obtained if a challenging but realistic goal was stated (e.g. increase walking by 30 minutes daily) and 1 point was obtained if the situation in which the change would be made was clearly and realistically stated (e.g. when going and returning from work). For PA, a third point could be obtained for filling out with whom one was planning to do the activity (e.g. 'with my partner' or 'alone').	Three points could be obtained for a stated PA goal, and two points could be obtained for a DI goal.
Use of the coping plan exercise	
A similar approach was used for use of the coping planning exercise, in particular how the participant planned to avoid or cope with a difficult situation in the first week of behaviour change. A dichotomous variable was created based on the participant's use of the coping planning component.	0: did not describe a coping plan, 1: described a coping plan

*Quality of the coping plans**	
Next, the content of the coping plan was coded to assess its quality. A coping plan was coded as ‘correct’ (scoring a 2) if a response was given that a) would facilitate the desired behaviour, and b) was feasible in the risk situations that were defined . If either or both these criteria were not met, 1 point was given to indicate an ‘incorrect plan’.	1: incorrect plan (nor criteria met)<br>2: correct plan
