# Supplementary material for: Intervention Use and Action Planning in a Web-Based Computer-Tailored Weight Management Program for Overweight Adults: Randomized Controlled Trial
Source: JMIR Res Protoc. 2014 Jul 23;3(3):e31. doi: 10.2196/resprot.2599 (PMC4129126; doi:10.2196/resprot.2599)
Supplement: Supplementary file 3 [file resprot_v3i2e31_app3.pdf]

CONSORT-EHEALTH Checklist V1.6.2 Report

(based on CONSORT-EHEALTH V1.6), available at [http://tinyurl.com/consort-ehealth-v1-6].

2599

Date completed
3/5/2013 6:45:32
by
Lenneke van Genugten

Intervention usage and action planning in a web-based computer-tailored weight management program for overweight adults: results from a RCT.

TITLE

1a-i) Identify the mode of delivery in the title
"Intervention usage and action planning in a web-based computer-tailored weight management program for overweight adults: results from a RCT. "
1a-ii) Non-web-based components or important co-interventions in title
"Intervention usage and action planning in a web-based computer-tailored weight management program for overweight adults: results from a RCT. "
1a-iii) Primary condition or target group in the title
"Intervention usage and action planning in a web-based computer-tailored weight management program for overweight adults: results from a RCT. "

ABSTRACT

1b-i) Key features/functionalities/components of the intervention and comparator in the METHODS section of the ABSTRACT
"Data were obtained in the randomised controlled effect evaluation trial in which the online computer-tailored intervention was compared to a website containing generic information about prevention of weight gain. The tailored intervention included self-regulation techniques such as personalised feedback, goal setting, action planning, monitoring and other techniques aimed at weight management. "
1b-ii) Level of human involvement in the METHODS section of the ABSTRACT
"Data were obtained in the randomised controlled effect evaluation trial in which the online computer-tailored intervention was compared to a website containing generic information about prevention of weight gain. "
1b-iii) Open vs. closed, web-based (self-assessment) vs. face-to-face assessments in the METHODS section of the ABSTRACT
"Participants were 539 overweight adults (mean age 46.9 years, mean BMI 28.03, 31.2% male, 11% low education level) recruited from the general population."

"Use of the intervention and its planning tools was derived from server registration data. Physical activity, fat intake, motivational factors and self-regulation skills were self-reported at baseline. "

1b-iv) RESULTS section in abstract must contain use data
Detailed information about the trial has also been reported in (van Genugten, 2012).

"Use of the tailored intervention decreased sharply after the first modules. Visiting the first tailored intervention module was more likely among participants with low levels of fat intake (OR=.77, 95%CI .62-.95) or planning for change in PA (OR 0.23, 95%CI .05-.97). Revisiting the intervention was more likely among participants high in restrained eating (OR2.45, 95%CI 1.12-5.43) or low in pro-active coping skills for weight control (OR.28, 95%CI .10-.76). The planning tools were used by 5-55% of the participants, but only 20-75% of the plans were of good quality. "
1b-v) CONCLUSIONS/DISCUSSION in abstract for negative trials
"This study showed that psychological factors such as self-regulation skills and action planning were associated with repeated use of an online, computer-tailored self-regulation intervention aimed at prevention of weight gain among adults being overweight. Use of the intervention was not optimal, with only limited numbers of participants who visited all the intervention modules. The use of the action and coping planning components of the intervention was mediocre and the quality of the generated plans was low, especially for the coping plans. It is important to identify how use of action planning and coping planning components in online interventions can be promoted and how the quality of plans generated through these tools can be improved. "

INTRODUCTION

2a-i) Problem and the type of system/solution
"The first aim of the present study was to identify factors that are associated with first and repeated use of an online weight gain prevention program for adults being overweight. The second aim of this study was to increase insight in amount and quality of use of the planning tools in the online interventions. "
"The intervention under study is an online, computer-tailored self-regulation program aimed at the prevention of weight gain among overweight (BMI 25-30) adults [15]. The computer-tailored intervention consisted of four modules which people could visit in a four to eight week period. "
2a-ii) Scientific background, rationale: What is known about the (type of) system
"Such interventions can be effective in improving a variety of behaviours and outcomes [5-8] especially when a planning tool is included [9]. However, there is also a large body of evidence to suggest that the use of online interventions is often low [10-12]. A steep decline in numbers of visitors to follow-up sessions is often observed, and non-optimal use or exposure to the intervention content may result in an underestimation of the effects that can be achieved with an online intervention [13]."

METHODS

3a) CONSORT: Description of trial design (such as parallel, factorial) including allocation ratio
"This study aims to answer the following 2 questions:
1.Which baseline demographic, motivational and behavioural factors and self-regulation skills are associated with first time and repeated use of an online computer-tailored self-regulation intervention aimed at preventing weight gain among overweight adults?
2.Do participants use the guided, open format tools for action planning and coping planning and, if so, what is the quality of the generated plans?
"

3b) CONSORT: Important changes to methods after trial commencement (such as eligibility criteria), with reasons
NA
3b-i) Bug fixes, Downtimes, Content Changes
NA
4a) CONSORT: Eligibility criteria for participants
"Participants enrolled in the study by filling out an online submission form. Subsequently, criteria for inclusion (25-60 years of age, BMI 25-30, ability to read and write in Dutch and easy access to the Internet) and exclusion criteria (pregnancy, following a diet prescribed by a dietician or physician, having a history of depression or eating disorder) were checked."
4a-i) Computer / Internet literacy
"..... ability to read and write in Dutch and easy access to the Internet) ....were checked."

4a-ii) Open vs. closed, web-based vs. face-to-face assessments:
"Participants were recruited from the general population through advertisements placed in local newspapers and flyers that were distributed door-to-door, in the waiting rooms of GPs and among the employees of four large companies."

"They also received an email in which they were asked to fill out the online baseline questionnaire (motivational factors, dietary intake, physical activity and self-regulation skills) online. Weight, height, waist circumference and skin fold thickness were measured at the hospital site where they also filled out the informed consent form. "

4a-iii) Information giving during recruitment
"They also received an email in which they were asked to fill out the online baseline questionnaire (motivational factors, dietary intake, physical activity and self-regulation skills) online. Weight, height, waist circumference and skin fold thickness were measured at the hospital site where they also filled out the informed consent form. "

4b) CONSORT: Settings and locations where the data were collected
"They also received an email in which they were asked to fill out the online baseline questionnaire (motivational factors, dietary intake, physical activity and self-regulation skills) online. Weight, height, waist circumference and skin fold thickness were measured at the hospital site where they also filled out the informed consent form. "

4b-i) Report if outcomes were (self-)assessed through online questionnaires
"Intention to prevent weight gain, perceived behaviour control, weight locus of control and restrained eating are potential determinants of intervention use and were assessed by online self-report at baseline. "

4b-ii) Report how institutional affiliations are displayed
Recruitment materials, assessment material/questionnaires and the intervention website all contained the university medical centers' logo.

5) CONSORT: Describe the interventions for each group with sufficient details to allow replication, including how and when they were actually administered

5-i) Mention names, credential, affiliations of the developers, sponsors, and owners
NA

5-ii) Describe the history/development process
detailed information about the development can be reac in (van Genugten, 2011)

The intervention was developed using the Intervention Mapping protocol [15, 21, 22]. The intervention's main objective was to prevent weight gain in overweight adults by inducing small changes (100 kcal/day) in energy balance-related behaviours. Examples of these changes include frequency and duration of physical activity and the intake of fat from several categories such as dairy, meat, cheese, sauce and gravy, snacks and sweetened drinks [23]. The intervention goals, methods and strategies were based on Self-Regulation Theory [24], motivational theories [25-27] and goal setting and action planning theories [26, 28].

5-iii) Revisions and updating
NA

5-iv) Quality assurance methods
Information was based on theory and evidence were possible. With respect to nutrition, we also applied information from the Netherlands Nutrition Center.

5-v) Ensure replicability by publishing the source code, and/or providing screenshots/screen-capture video, and/or providing flowcharts of the algorithms used
See (van Genugten 2012) en appendices.

5-vi) Digital preservation
Currently, the intervention s not accessible for the public. Those who are interested can ask the author for the link, a username and a password.

5-vii) Access
"All randomised participants received a login name and password by e-mail, to login to their assigned intervention program. Participants were asked to visit the websites at least three/four times during a two month period. They received bi-weekly e-mail reminders to (re-) visit the intervention. Six months after completion of the intervention period, participants were asked by e-mail to fill out the online questionnaire again and their anthropometrics were assessed at the hospital site. Phone calls were made to participants who did not respond by e-mail. Participants who filled out the questionnaire and had their anthropometrics measured at the six month follow-up, received a gift voucher of €10. "

5-viii) Mode of delivery, features/functionalities/components of the intervention and comparator, and the theoretical framework
"The intervention was developed using the Intervention Mapping protocol [15, 21, 22]. The intervention's main objective was to prevent weight gain in overweight adults by inducing small changes (100 kcal/day) in energy balance-related behaviours. Examples of these changes include frequency and duration of physical activity and the intake of fat from several categories such as dairy, meat, cheese, sauce and gravy, snacks and sweetened drinks [23]. The intervention goals, methods and strategies were based on Self-Regulation Theory [24], motivational theories [25-27] and goal setting and action planning theories [26, 28]. "

More information can be found in (van Genugten, 2011)

5-ix) Describe use parameters
"Participants were asked to visit the websites at least three/four times during a two month period. They received bi-weekly e-mail reminders to (re-) visit the intervention. "

"In order to deliver the self-regulation strategies in a timely fashion, the intervention consisted of four modules, each to be visited one week after the previous one, to work through all steps of self-regulation (goal setting, active goal pursuit and evaluation [24]). Completion of all modules would take about 90 minutes. "

5-x) Clarify the level of human involvement
Participants could call the researchers or send them an e-mail when they needed technical assistance.

5-xi) Report any prompts/reminders used
"They received bi-weekly e-mail reminders to (re-) visit the intervention. "

5-xii) Describe any co-interventions (incl. training/support)
NA

6a) CONSORT: Completely defined pre-specified primary and secondary outcome measures, including how and when they were assessed

"An objective measure of intervention usage was obtained by retrieving the log-in data from the intervention server registrations, which registered how often each participant logged on to the program and which intervention modules they visited (0-3 for GI, 0-4 for TI). First, a dichotomous 'never-ever' score was created, with 0 indicating 'never visited' and 1 indicating 'visited at least once' (sum score > 1). For those who visited at least one module (sum score > 1), a dichotomous score was made for 'revisiting' (visited first module only: 0, also visited later modules: 1)."

"Information about the use of the action planning and coping planning components and the quality of the plans developed by the participants was also obtained from the intervention server registration, where the plans that had been written were stored. Two dichotomous variables were made, indicating whether or not people chose to make a change in dietary intake and/or physical activity. A dichotomous variable was created for use of the action planning component, coded as 0: no plan, 1: a plan. Then, the quality of the goal was determined, by scoring the text that was written in the text boxes in the program. For this text, one point was obtained if a challenging but realistic goal was stated (e.g. increase walking by 30 minutes daily) and 1 point was obtained if the situation in which the change would be made was clearly and realistically stated (e.g. when going and returning from work). For PA, a third point could be obtained for filling out with whom one was planning to do the activity (e.g. 'with my partner' or 'alone'). Therefore, three points could be obtained for a stated PA goal, and two points could be obtained for a DI goal.
A similar approach was used for use and quality of the coping plans, in particular how the participant planned to avoid or cope with a difficult situation in the first week of behaviour change. A dichotomous variable was created based on the participant's use of the coping planning component (0: did not describe a coping plan, 1: described a coping plan). Next, the content of the coping plan was coded to assess its quality. A coping plan was coded as 'correct' (scoring a 2) if a response was given that a) would facilitate the desired behaviour, and b) was feasible in the risk situations that were defined (van Osch, Lechner et al. 2008). If either or both these criteria were not met, 1 point was given to indicate an 'incorrect plan'.
"

6a-i) Online questionnaires: describe if they were validated for online use and apply CHERRIES items to describe how the questionnaires were designed/deployed
All questionnaires have been validated. Most have been applied online before, nut none is validated for online use.
6a-ii) Describe whether and how "use" (including intensity of use/dosage) was defined/measured/monitored
See question 6a.
6a-iii) Describe whether, how, and when qualitative feedback from participants was obtained
NA
6b) CONSORT: Any changes to trial outcomes after the trial commenced, with reasons
NA
7a) CONSORT: How sample size was determined
7a-i) Describe whether and how expected attrition was taken into account when calculating the sample size
See (van Genugten, 2012)
7b) CONSORT: When applicable, explanation of any interim analyses and stopping guidelines
NA
8a) CONSORT: Method used to generate the random allocation sequence
NA
8b) CONSORT: Type of randomisation; details of any restriction (such as blocking and block size)
Stratified (gender) block randomization (10 participants/ block). Also see (van Genugten, 2012)
9) CONSORT: Mechanism used to implement the random allocation sequence (such as sequentially numbered containers), describing any steps taken to conceal the sequence until interventions were assigned
computerized random allocation. Also see (van Genugten, 2012)
10) CONSORT: Who generated the random allocation sequence, who enrolled participants, and who assigned participants to interventions
Computer generated sequence. Participants were enrolled by the first author, who also assigned them to their intervention.
11a) CONSORT: Blinding - If done, who was blinded after assignment to interventions (for example, participants, care providers, those assessing outcomes) and how
11a-i) Specify who was blinded, and who wasn't
Participants and those who took the measurements were blinded.
11a-ii) Discuss e.g., whether participants knew which intervention was the "intervention of interest" and which one was the "comparator"
They did not know to which condition they were assigned. They might have guessed it based on the length and interaction in the interventions.
11b) CONSORT: If relevant, description of the similarity of interventions
"Those randomly assigned to the generic information (GI) condition had access to a website that provided generic information on weight management. This information could be worked through in three modules. The first module provided generic information about the goal of weight gain prevention, such as health risks associated with weight gain. The second module provided information about possible behaviour changes. The third module provided general information about a healthy diet and safe physical activity. The visual design of the website was the same as the tailored intervention and participants received the same reminders as the TI group. "
12a) CONSORT: Statistical methods used to compare groups for primary and secondary outcomes
"Descriptive statistics were used to describe the study population. T-test and chi-square tests were used to compare the two intervention groups on baseline demographic, behavioural and psychological factors. Logistic regression analyses were applied to study participant predictors of first intervention visit and follow-up visits. In order to identify the best predictors of usage, a backward elimination (likelihood ratio) procedure was used. Independent variables were age, education, sex, BMI, fat intake, physical activity, intention and perceived behavioural control for WGP, weight locus of control, restrained eating, monitoring of weight, action planning for change in DI and PA and pro-active coping skills as assessed at baseline. Descriptive statistics were used to describe the usage of the self-regulation components and the quality of the plans. "
12a-i) Imputation techniques to deal with attrition / missing values
NA
12b) CONSORT: Methods for additional analyses, such as subgroup analyses and adjusted analyses
NA

RESULTS

13a) CONSORT: For each group, the numbers of participants who were randomly assigned, received intended treatment, and were analysed for the primary outcome
"In total, 630 people completed the online registration, and 539 initially participated by completing the baseline questionnaire and/or coming in for anthropometric measurements. Subsequently, the anthropometric measures of 480 people were taken at baseline, and 313 of the 480 returned for measurements 6 months after the intervention (drop-out rate of 39.5%). A total of 516 participants completed the baseline questionnaire and 361 participants filled out the six-month follow-up questionnaire (drop-out rate of 33%). "

See table 1, and (van Genugten 2012)

13b) CONSORT: For each group, losses and exclusions after randomisation, together with reasons
See (van Genugten, 2012)
13b-i) Attrition diagram
See (van Genugten, 2012)
14a) CONSORT: Dates defining the periods of recruitment and follow-up
"The study was conducted between March 2009 and October 2010. "
See (van Genugten, 2012)
14a-i) Indicate if critical "secular events" fell into the study period
NA
14b) CONSORT: Why the trial ended or was stopped (early)
NA
15) CONSORT: A table showing baseline demographic and clinical characteristics for each group
See table 1 and (van Genugten, 2012)
15-i) Report demographics associated with digital divide issues
See table 1 and (van Genugten, 2012)
16a) CONSORT: For each group, number of participants (denominator) included in each analysis and whether the analysis was by original assigned groups
16-i) Report multiple "denominators" and provide definitions
See tables and (van Genugten, 2012)
16-ii) Primary analysis should be intent-to-treat
Reported in (van Genugten, 2012)
17a) CONSORT: For each primary and secondary outcome, results for each group, and the estimated effect size and its precision (such as 95% confidence interval)
Yes, e.g. "Logistic regression analysis (table 2) showed that first time usage of the tailored intervention is more likely among participants with a lower level of physical activity (OR=0.98, 95% CI .96-.999), lower action planning for PA (OR=0.23, 95%CI .06-.9) and lower fat intake (OR=0.77, 95%CI .62-.95) at baseline. Re-visiting the tailored intervention was more likely among people with fewer pro-active coping skills (OR=0.28, 95% CI 0.10-0.76) and higher levels of restrained eating (OR=2.45, 95% CI 1.11-5.43) at baseline. "
17a-i) Presentation of process outcomes such as metrics of use and intensity of use
Yes, e.g. figure 1 and table 3.
17b) CONSORT: For binary outcomes, presentation of both absolute and relative effect sizes is recommended
NA.
18) CONSORT: Results of any other analyses performed, including subgroup analyses and adjusted analyses, distinguishing pre-specified from exploratory
This whole paper addresses subgroup analyses.
18-i) Subgroup analysis of comparing only users
See table 2 and 3.

"A disadvantage of this study is the inclusion of the intervention group only in some analyses, possibly resulting in an biased sample of users. "
19) CONSORT: All important harms or unintended effects in each group
NA
19-i) Include privacy breaches, technical problems
NA
19-ii) Include qualitative feedback from participants or observations from staff/researchers
NA

DISCUSSION

20) CONSORT: Trial limitations, addressing sources of potential bias, imprecision, multiplicity of analyses
20-i) Typical limitations in ehealth trials
"One of this study's strengths is its use of objective information to assess the level of usage of the website and the goal setting and coping planning tools. Additionally, we were able to link intervention use to personal characteristics, making it possible to describe characteristics of users and non-users. Also, BMI was measured in an objective way, but other correlates of intervention use were based on self-report. A disadvantage of this study is the inclusion of the intervention group only in some analyses, possibly resulting in an biased sample of users. Furthermore, it was not possible to compare the open-ended planning format to a closed-ended planning format. "
21) CONSORT: Generalisability (external validity, applicability) of the trial findings
21-i) Generalizability to other populations
As this were participants from the general population and they were 'free' to use the intervention as they pleased, we think the results reflect a natural proces. More information about generalization can be read in (van Genugten 2012.)
21-ii) Discuss if there were elements in the RCT that would be different in a routine application setting
NA
22) CONSORT: Interpretation consistent with results, balancing benefits and harms, and considering other relevant evidence
22-i) Restate study questions and summarize the answers suggested by the data, starting with primary outcomes and process outcomes (use)
"In this study, we examined usage and predictors of usage of an online computer-tailored weight gain prevention intervention for overweight adults. Initial use of the TI was high (93%), but only 27% of the participants visited three modules and 15% completed all four modules. Initial visit of the control condition was lower (82%), but completion of all three modules was higher (46%) compared to the TI condition. Use of the first tailored intervention module was more likely among participants who had a lower fat intake, lower physical activity, and lower action planning for PA at baseline. Repeated use of the tailored information condition was more likely among participants with higher levels of restrained eating and who had a lower score on pro-active coping skills at baseline. Of those who used the tailored intervention, 55% stated a goal for a change in DI and 15% for a change in PA. Only 28% made a coping plan for DI and 5% for PA. About half of the written goals and plans were of good quality."
22-ii) Highlight unanswered new questions, suggest future research

"Unlike other studies [10, 11, 42], use and repeated use of the intervention were not associated with motivational factors and socio-demographic characteristics. This may indicate that the program was equally appealing to people with higher and lower educational levels, higher and lower self-efficacy and higher or lower intention for WGP. In order to increase use of online intervention, it is important to learn more about the relation between participant characteristics and usage. "

"It is, therefore, of high importance to identify how the usage quality of action and coping plans in online interventions can be improved. "

" It is important to identify how overall use of the intervention can be improved, as well as use and quality of action planning and coping planning components. "

Other information

23) CONSORT: Registration number and name of trial registry

NTR1862; http://apps.who.int/trialsearch/trial.aspx?trialid=NTR1862

24) CONSORT: Where the full trial protocol can be accessed, if available

(van Genugten, 2011)

25) CONSORT: Sources of funding and other support (such as supply of drugs), role of funders

This work was supported by a grant from ‘The Netherlands Organisation for Health Research and Development’ (ZonMW) [6130.0025]

X26-i) Comment on ethics committee approval

"The Medical Ethics Committee of the Erasmus MC, University Medical Centre Rotterdam, issued a declaration of no objection for the study, and all participants gave their written informed consent. "

x26-ii) Outline informed consent procedures

"Weight, height, waist circumference and skin fold thickness were measured at the hospital site where they also filled out the informed consent form."

X26-iii) Safety and security procedures

NA

X27-i) State the relation of the study team towards the system being evaluated

There are no conflicts of interest.
